# Organ-specific remodeling of the Arabidopsis transcriptome in response to spaceflight

**DOI:** 10.1186/1471-2229-13-112

**Published:** 2013-08-07

**Authors:** Anna-Lisa Paul, Agata K Zupanska, Eric R Schultz, Robert J Ferl

**Affiliations:** 1 Department of Horticultural Sciences, Program in Plant Molecular and Cellular Biology, University of Florida, Gainesville, FL, 32611, USA; 2 Interdisciplinary Center for Biotechnology, University of Florida, Gainesville, FL, 32611, USA

## Abstract

**Background:**

Spaceflight presents a novel environment that is outside the evolutionary experience of terrestrial organisms. Full activation of the International Space Station as a science platform complete with sophisticated plant growth chambers, laboratory benches, and procedures for effective sample return, has enabled a new level of research capability and hypothesis testing in this unique environment. The opportunity to examine the strategies of environmental sensing in spaceflight, which includes the absence of unit gravity, provides a unique insight into the balance of influence among abiotic cues directing plant growth and development: including gravity, light, and touch. The data presented here correlate morphological and transcriptome data from replicated spaceflight experiments.

**Results:**

The transcriptome of *Arabidopsis thaliana* demonstrated organ-specific changes in response to spaceflight, with 480 genes showing significant changes in expression in spaceflight plants compared with ground controls by at least 1.9-fold, and 58 by more than 7-fold. Leaves, hypocotyls, and roots each displayed unique patterns of response, yet many gene functions within the responses are related. Particularly represented across the dataset were genes associated with cell architecture and growth hormone signaling; processes that would not be anticipated to be altered in microgravity yet may correlate with morphological changes observed in spaceflight plants. As examples, differential expression of genes involved with touch, cell wall remodeling, root hairs, and cell expansion may correlate with spaceflight-associated root skewing, while differential expression of auxin-related and other gravity-signaling genes seemingly correlates with the microgravity of spaceflight. Although functionally related genes were differentially represented in leaves, hypocotyls, and roots, the expression of individual genes varied substantially across organ types, indicating that there is no single response to spaceflight. Rather, each organ employed its own response tactics within a shared strategy, largely involving cell wall architecture.

**Conclusions:**

Spaceflight appears to initiate cellular remodeling throughout the plant, yet specific strategies of the response are distinct among specific organs of the plant. Further, these data illustrate that in the absence of gravity plants rely on other environmental cues to initiate the morphological responses essential to successful growth and development, and that the basis for that engagement lies in the differential expression of genes in an organ-specific manner that maximizes the utilization of these signals – such as the up-regulation of genes associated with light-sensing in roots.

## Background

The completion of the International Space Station (ISS), including the installation of experiment hardware and the presence of a regular crew complement, presents enormous opportunity to examine the longer term effects of spaceflight and microgravity on living systems. ISS capabilities now include stable orbital environment, flexible-environment growth chambers, on orbit imaging, functional laboratory-bench areas, crew time for harvest, and a facile, reliable sample storage and return strategy [1-3]. Given these capabilities, the 2010 NRC Decadal Survey, Recapturing a Future for Space Exploration: Life and Physical Sciences Research for a New Era [4] strongly encouraged the application of molecular biology technologies to ISS studies to address fundamental questions of plant growth and development in spaceflight, in the absence of unit gravity, which is considered a major environmental force shaping plant evolution.

Plants have a long and international history in spaceflight research (recent reviews include: [5-10]), and because of the relationship between gravity and plant architecture [11], plants are considered important tools for discovery of gravity-related biological phenomena [7]. Yet the cultivation of plants within spaceflight vehicles presented numerous physical and engineering challenges that complicated interpretation of fundamental plant responses. For example, the management of root-zone water and aeration is affected by the lack of convection and related boundary layer issues, along with the unique behavior of water and capillary action in microgravity, and the attendant engineering designed to ameliorate the impact of these issues is an important feature of spaceflight experiments [10,12]. Early experiments with plants highlighted the need for understanding and overcoming these technical limitations, while nonetheless establishing that near typical plant growth can occur in space. Many studies indicated that as engineering challenges were overcome, plant growth and development approached terrestrial norms [6,9,13].

Even as engineering challenges have been overcome, investigations have shown that plants in various growth systems do respond to spaceflight with modified gene expression patterns. *Arabidopsis* seedlings in completely sealed canisters showed changes in gene expression consistent with cell wall modifications, while *Arabidopsis* cell cultures demonstrated a very different gene expression response from that of seedlings [14-16]. Plants in a single-celled stage, such as the gravity-sensitive spores of fern *Ceratopteris richardii* also respond to spaceflight with changes in gene expression patterns [17,18]. Conversely, wheat leaves, using limited arrays, showed few significant changes in transcriptional profiles between spaceflight and ground environments [19].

The work reported here is a biologically replicated molecular analysis of 12-day-old *Arabidopsis* plants grown on phytagel plates within the Advanced Biological Research System (ABRS). The designation of this flight experiment was TAGES, which is an acronym for Transgenic Arabidopsis Gene Expression System. The use of plates within the ABRS created a benign culture environment that minimized hardware-induced stress factors. With the roots coursing along the surface of the media, problems with root-zone aeration and water and nutrient delivery were eliminated while the ABRS provided controlled lighting, temperature, and air quality. Leaves, hypocotyls and roots all differ in their gene expression responses to spaceflight, and spaceflight appears to primarily affect several hormone signaling pathways, which may drive extensive cell wall remodeling especially in roots.

## Results

### Access to facilities on the ISS allowed facile spaceflight growth, imaging, and harvest of Arabidopsis

**Figure 1:**
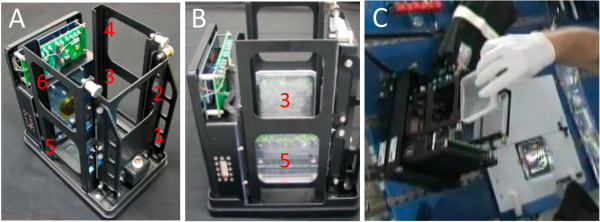
**The GFP Imaging System (GIS). (A)** A photograph of the GIS hardware with the 6 slots for plates. Each slot is assigned a number (shown in red) to identify plate position: positions 1, 3, and 5 are on the bottom tier, and positions 2, 4, and 6 are in the top tier. The plate in position 1 is situated opposite the camera, which can collect both fluorescent- and white-light images of plate 1. **(B)** A side view of the GIS shows the configuration of plates 3 and 5. **(C)** On orbit, plates can be easily removed for harvest at the Maintenance Work Area (MWA), which is a surface in the US Lab of the ISS that is equipped for biological science, including a docking station for the GIS and a photogrid for the plates. The image shows an astronaut removing a plate from the GIS as well as a plate attached to the photo grid to the right of the GIS.

**Figure 2:**
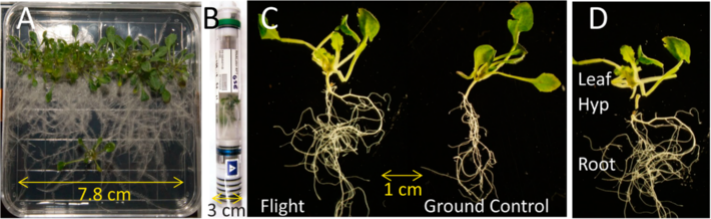
**In flight harvests for transcriptome analyses. (A)** A representative upper-tier plate (plate 6 from run 1B). **(B)** A Kennedy Space Center Fixation Tube (KFT) photographed after it had been returned to authors. Plant material can be seen in the central chamber. **(C)** Representative individual flight (left) and ground control (right) plants after they have been removed from the KFT. **(D)** An example of how individual plants were separated into leaf, hypocotyl, and root fractions.

Growth of *Arabidopsis* on standard laboratory Petri plates was conducted within the ABRS using the GFP Imaging System (GIS) (Figure 1A, B - see Methods), which provided straightforward crew access for harvesting (Figure 1C). The plants exhibited robust growth (Figure 2A), and after 12 days were harvested to RNAlater in KSC Fixation Tubes (KFTs, Figure 2B). The loaded KFTs were frozen for storage on orbit and eventual return to earth. After return, the spaceflight plants (FLT), together with ground control plants (GC) (Figure 2C), were recovered from RNAlater and dissected into roots, leaves, and hypocotyls for analysis (Figure 2D). Ground control experiments were conducted using identical ABRS hardware and an environmental chamber programmed to ISS environmental conditions. Both flight and ground control plants were harvested after 12 days of growth to KFTs, and ground control samples were collected parallel to every spaceflight sample.

### The transcriptome of Arabidopsis demonstrated organ-specific changes in response to spaceflight

**Figure 3:**
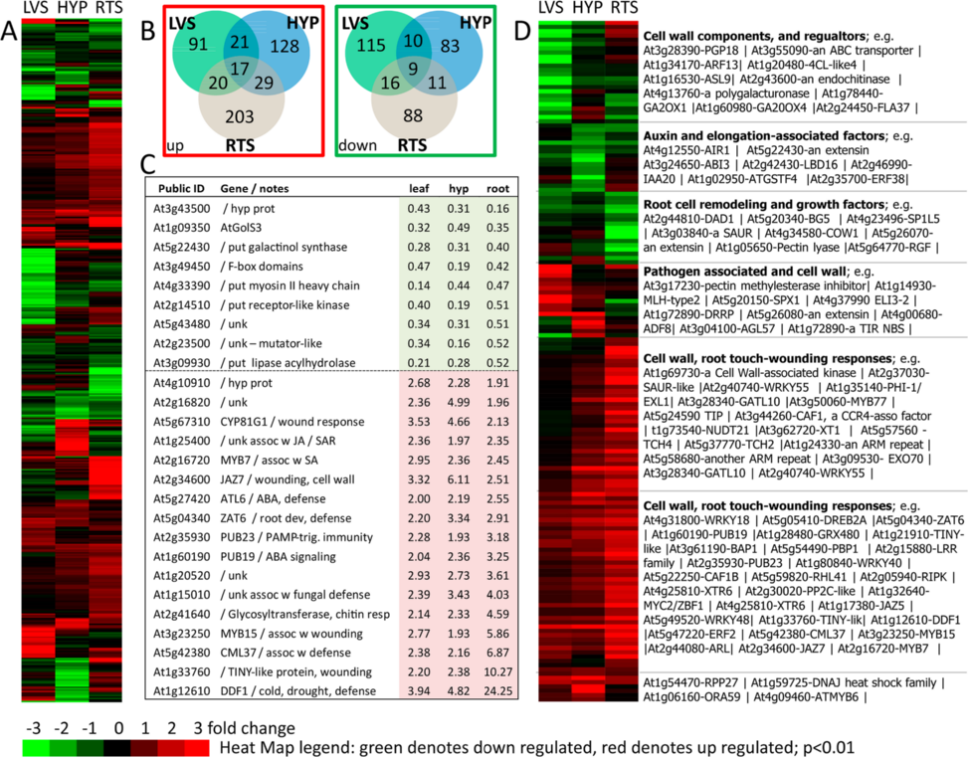
**Differentially expressed genes in response to the spaceflight environment. (A)** The hierarchical clustering of all 480 genes with statistically significant (p < 0.01) differential expression in the spaceflight environment by at least 1.9-fold in at least one of the three organs (LVS – Leaves; HYP – Hypocotyls; RTS – Roots). **(B)** A pair of Venn diagrams that illustrates the organ-specific gene expression patterns of up (red box, left) and down (green box, right) among leaves (green), hypocotyls (blue), and roots (tan). **(C)** A list of the 26 genes coordinately up or down regulated in all organs. The Public ID number is annotated with a brief description, and the corresponding fold-change values are shown in the columns designated for each organ. **(D)** A hierarchical clustering of 158 genes from **(A)**, which have an association with cell wall remodeling and cell expansion, pathogen or wounding responses, and growth hormone signal transduction. A partial annotation of genes representative of each cluster is given to the right of the graphic. A fully annotated version of **(D)** is presented in Additional file 3: Figure S1. Green indicates down-regulated genes and red indicates up-regulated genes. Heat-map legend values are in log_[2]_. Hierarchical clustering methods used on the graphics were after [20].

There were 480 genes that showed significant (p < 0.01) differential expression by at least 1.9-fold (Figure 3A, Additional file 1) and 58 by more than 7-fold (Additional file 2) in any organ; yet leaves, hypocotyls, and roots each displayed unique patterns of gene expression in response to spaceflight. Figure 3B provides a pair of Venn diagrams that illustrates the distribution patterns of differentially expressed genes among leaves (green circle), hypocotyls (blue circle), and roots (tan circle).

There were 26 genes that were coordinately expressed in roots, hypocotyls, and leaves by at least 1.9-fold in all organs; 17 of these genes up-regulated and 9 down-regulated (Figure 3C). All but one of the coordinately up-regulated genes with known functions are associated with plant cell wall remodeling and defense response. Only 6 of the 9 coordinately down-regulated genes have a known function; three are also associated with cell wall remodeling and defense response, and the other three are generally associated with signal transduction that impacts growth.

Most of the genes differentially expressed in response to spaceflight are not coordinately expressed among organs (Figure 3A, B and D); however, in each organ there remains a strong tendency for the involvement of defense and cell remodeling genes. Figure 3D shows a subset of 158 genes chosen from the 480 shown in Figure 3A, which are genes that have been associated with various aspects of cell wall remodeling and cell expansion, pathogen and wounding responses, or growth hormone signal transduction. Each cluster is annotated with a few representative genes; a fully annotated version can be found in Additional file 3. As in the set of coordinately expressed genes, the down-regulated genes were largely associated with a number of factors regulating cell elongation and hormone signal transduction. The up-regulated genes were closely associated with cell wall remodeling and touch, wounding, and pathogen responses. Most of the highly up-regulated genes were represented in roots, including 20 of the 31 genes induced by more than 7-fold (Additional file 2).

### Selected transcriptional responses to spaceflight were shown to be consistent among distinct flight experiments

**Figure 4:**
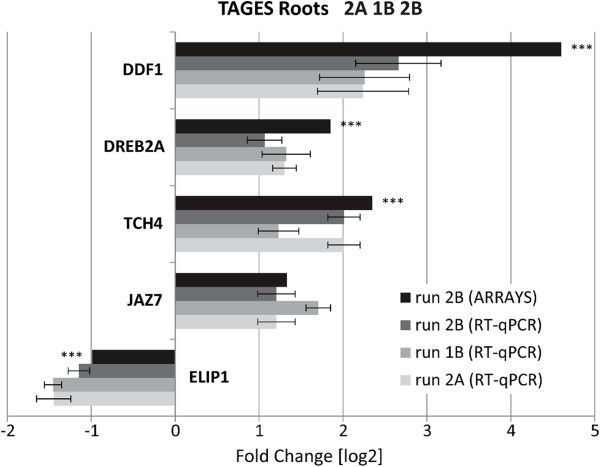
**Quantitative analyses across three separate spaceflight experiments.** Quantitative RT-qPCR analyses were used to check the consistency of gene expression in root RNA across three separate TAGES flight experiments: Run 1B, Run 2A, and Run 2B. Runs 1B, 2A, and 2B were composed of identical *Arabidopsis* lines and grown under identical conditions, although in three distinct windows of time on the ISS (see Methods). The fold-change between flight and ground control is presented for five genes showing statistically significant (p < 0.01) differential expression in the microarrays conducted for the Run 2B plants (black). The RT-qPCR fold-change values (in log_[2]_) for each gene are presented below the array values in gradients of gray bars: Run 2B (dark gray), Run 1B (medium gray), Run 2A (light gray). Error bars for each RT-qPCR set indicate Standard Deviation with an n = 3 of biological replicates. The asterisks (***) associated with array data bars indicate p ≤0.005; otherwise, p ≤0.01. The public ID numbers for the genes presented are: DDF1 (At1g12610), DREB2A (At5g05410), TCH4 (At5g57560), JAZ7 (At2g34600), ELIP1 (At3g22840).

RT-qPCR data for five selected target genes were obtained from two additional, comparable flight experiments. The genes DDF1, DREB2A, TCH4, JAZ7 and ELIP1 were initially chosen to provide quantitative support for the Run 2B array data, being selected on the basis of their high differential expression in those arrays and functional interest. This same set of genes was later used to evaluate transcriptional trends displayed by the roots of plants in the 1A and 2A flight experiments. DDF1, DREB2A, TCH4, and JAZ7 were all similarly induced in the three spaceflight growth time frames, while ELIP1 was similarly repressed (Figure 4). These data illustrated the repeatability of the spaceflight response across two separate launches and three distinct ISS growth experiments conducted months apart.

### Changes in auxin-mediated signaling occurred over the course of plant development with the GFP reporters

**Figure 5:**
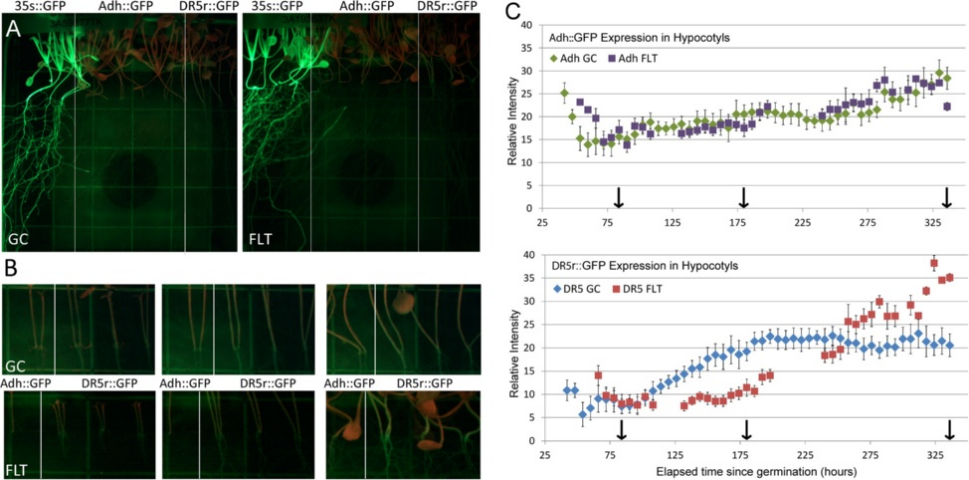
**GFP reporter gene images from Run 3A, Plate 1. (A)** Three GFP reporter gene lines were planted on each plate: 35s::GFP (left), Adh::GFP (center) and DR5r::GFP (right). The ground control plate (GC) is shown on the left and the corresponding flight plate (FLT) on right. Both were 14.5-days old at the end of the experiment. The constitutive expression of the GFP reporter in the 35s::GFP plants is visible in both images. There was no apparent GFP expression induced by the Adh promoter (Adh::GFP), although the DR5r::GFP plants show differential GFP expression in hypocotyls. **(B)** Enlarged views of Adh::GFP and DR5r::GFP hypocotyls are shown for three developmental time points: 3.5, 7.5, and 14 days. These time points correspond to the arrows along the x-axis in the graphs of **(C)**. **(C)** Quantification of green fluorescence in Adh::GFP and DR5r::GFP hypocotyls for ground control and flight plants over time. The graphs plot the average GFP value in the Adh::GFP and DR5r::GFP hypocotyls at each time point. Ground control (GC) values are presented in diamonds (Adh-green, DR5r::GFP-blue) and flight (FLT) values as squares (Adh-purple, DR5r::GFP-red). The y-axis provides green channel intensity in arbitrary units, and the x-axis shows the elapsed hours since germination was initiated. Images were collected every 6 hours. Missing FLT values on the graph are due to missing orbital images. Each value is the average of the scan of the same set of hypocotyls captured at each time point; error bars reflect standard error of the mean: n = 5 for GC; n = 7 for FLT. These images were identically treated with respect to background noise subtraction and adjustments of brightness and contrast to enhance visibility of hypocotyls. Visibility adjustments of these images do not affect the quantitative data collected through ImageJ from the original images and those presented in the graph.

A DR5r:GFP reporter gene line was imaged in Run 3A over time to evaluate potential changes in auxin-related gene expression patterns through development on orbit. The three reporter gene lines shown in Figure 5A and 5B are delineated by white lines: positive control 35s:GFP plants at left, Adh:GFP plants in the center, and DR5r:GFP plants at right. The constitutive expression of GFP in the 35s:GFP plants was apparent. No apparent differential GFP expression between flight and ground control was induced in the Adh:GFP plants. However, the DR5r:GFP plants showed differential GFP expression in the hypocotyls in spaceflight compared with those of ground controls over the duration of the flight. In the first few days after germination on orbit, the DR5r:GFP levels were similar for flight plants and the comparable ground controls. As development progressed through day 5, the relative expression of GFP in the ground controls exceeded that of the flight plants. Later, beginning at day10, the flight plants showed an increase of expression and exceeded expression levels of the ground controls, which remained static after day 6. Examples of images from these time frames, along with graphical representation of the green intensity values are shown in Figure 5b and 5C.

## Discussion

Completion of the ISS, together with operational hardware, effective experimentation strategies, and sample return protocols, has allowed for the first time a complete, replicated, organ-specific transcriptome analysis for physiological adaptation of *Arabidopsis* to spaceflight in laboratory-typical plant growth conditions. The present experiments compare spaceflight plants to plants grown on the ground in robustly replicated conditions, using ground hardware and procedures identical to those of the ISS. These experiments do not separate gravity out as single factor, but rather consider spaceflight in all of its collective inputs. A 1g centrifuge on orbit would allow experiments to begin to separate gravity from the other environmental factors of spaceflight (e.g. [21-23]), provided that the orbital centrifuge was large enough to eliminate gravity gradients within the plants. It is unlikely, however, that artificial gravity will be possible on any likely orbital or transit vehicles, so there is scientific and operational value in examining the collective effects of spaceflight on terrestrial biology, and especially plants if they are to be used for supplementing life support in extended orbital and long distance transit vehicles such as those envisioned for trips to Mars [24-26].

There were 480 genes observed to be differentially expressed in spaceflight versus ground control plants. The observation that only 480 genes were affected is remarkable, given that spaceflight plants and ground controls were operationally separated by 230 miles of altitude, 17,000 mph of velocity, and the other attendant potential impacts of orbital spaceflight, indicating that spaceflight environments have become extremely well controlled. Yet those 480 genes reveal a specific, rather robust, and in some ways unexpected physiological adaptation to spaceflight.

Leaves, roots, and hypocotyls engage different genes but adopt similar strategies to adapt to the spaceflight environment. Although unexpected, the major thread that connects the responses in these organs is that many of the differentially expressed genes are associated with touch, wound response, and cell wall remodeling, as well as the hormone signaling pathways that govern these processes.

In leaves, genes involved with hormone signaling, such as ABC transporters and auxin response factors [27,28], are highly repressed, as are several genes encoding cell wall associated proteins, including an endochitinase, polygalacturonase, xylan glucuronosyltransferase, an arabinogalactan precursor, and a lateral organ boundary-domain containing protein [29,30]. The highly up-regulated genes unique to leaves are primarily associated with defense typical of leaf herbivory and pathogen response [31,32], and some calcium and phosphate metabolism signaling that may be associated with wounding and gravity sensing [33,34]. At least one gene, PGP18, has been associated with both light signal perception and cell wall remodeling [35]. While this gene is highly up-regulated in leaves, the same PGP18 is highly repressed in roots.

In hypocotyls, genes associated with cell expansion are strongly represented, which is consonant with the fact that hypocotyl growth is primarily dictated by cell expansion [30,36]. A large component of cell expansion is the remodeling of the cell wall to accommodate the change in cell shape and volume [36,37]. Auxin-regulated genes are the most abundant, with dozens of highly differentially expressed genes similarly represented in an auxin-treated hypocotyl transcriptome [30] and other auxin- and BL-induced transcriptomes [38]. A morphometric feature of these spaceflight *Arabidopsis* hypocotyls is that they are shorter than the comparable ground control plants [39]. Only a few of the induced representatives are regulated through the AuxRE element, with four SAUR-like genes containing AuxRE motifs in their promoters up-regulated by 1.5- and 4-fold in hypocotyls. DR5r:GFP shows a differential expression profile in the spaceflight hypocotyls compared with ground controls, being repressed early but induced later in hypocotyl development, indicating a potentially complex auxin-related response in spaceflight.

In roots, cell wall remodeling dominates the tactical response to spaceflight, reflecting morphological differences that have been observed [39]. The roots of flight plants are smaller than their ground control cohorts, and in the case of the Col-0 ecotype, there are fewer lateral roots [39]. One gene family that contributes to both root length and the distribution and abundance of lateral roots contains the armadillo (ARM) repeat proteins [40]. In spaceflight, three members of this family are up-regulated, and one of them is up-regulated in all three organs and highly induced in roots. The flight roots also show clear signs of negative phototropism and strong skewing [39]. There is likely a strong connection between this size and skewing phenomenon and the large collection of genes involved in pathways that remodel cell walls, including the highly differentially expressed DDF1, CAF1, TCH4, COW1, EXT-like, and SP1L5 [41-46] genes. Several of the highly induced genes in roots were also identified as binding targets for HY5, a transcription factor associated with numerous photoreceptors [47,48]. The gene RHL41 (Responsive To High Light 41), which is up-regulated three-fold in roots, is a well-known HY5 target. However, the up-regulated genes EXO, XT1, NUDT21 and WRKY48, which are typically associated with touch and wounding, have also been identified as HY5 targets genes [48]. It is possible that the induction of HY5 partners, and other light responsive genes in roots, is part of a strategy to maximize utilization of the primary tropic signals available to plants in the absence of gravity: light and touch.

## Conclusions

There are four major abiotic cues involved in directing terrestrial plant growth and development: gravity, light, water, and touch (recent reviews: [7,34,49-52]). The opportunity to examine the strategies of environmental sensing in spaceflight provides a unique insight into the balance among these cues during the course of plant development. Some features of root growth thought to be gravity-dependent are not [39,53], and some features of phototropism [21,54] and signal transduction [17,18,55] would not have been revealed had it not been for the ability to remove the influence of gravity from the analyses. And while spaceflight may be more complex than simply the removal of gravity, gravity is certainly largely absent during the growth of these orbital plants.

Plant growth and development are governed by a vast and complex network of genes. The correlations in morphological and molecular data from spaceflight offer a unique insight into growth and development, particularly in regard to processes presumed to be affected by gravity. There is a rich history of plant biology using the spaceflight environment that may lead to a greater understanding of the role of gravity [9]. Additionally, the contemporary access to the ISS, along with new orbital habitats and hardware for plant experiments, will enable researchers to design a new generation of replicated experiments with which to explore these phenomena. It is likely that an understanding of responses to spaceflight will be refined as specific cell types are examined, and it is likely that the specific roles of gravity in spaceflight responses will be elaborated with facile access to orbital centrifuges. The result will be a much deeper understanding of plant growth in support of human spaceflight missions, as well as a deeper appreciation of the physiological adaptations of life forms to extra-terrestrial environments.

## Methods

### Independent spaceflight launches and replicate experiments

The data presented here are taken from several independent experiments launched to the ISS: STS-129, November 16, 2009 (Run 1B: 12/3/2009–12/15/2009); STS-130, February 8, 2010 (Run 3A: 2/21/2010–3/7/2010); and STS-131, April 5, 2010 (Runs 2A: 4/9/2010–4/21/2010, and 2B: conducted from 4/21/2010–5/3/2010, and returned on STS-132). Runs 1B, 2A, and 2B provided material for the gene expression analyses; each of these independent experiments was composed of three identical biological replicates composed of 12 day old plants. Run 3A was an imaging experiment that ran for 14 days; those plants were not included in the transcriptome analyses.

Each flight experiment (1B, 2A, 2B, 3A) had its own comparable, high fidelity ground control. Each ground control was housed in the Orbital Environmental Simulator (OES) at the Kennedy Space Center (KSC) and conducted in ABRS hardware units identical to the one installed on the ISS. The ground controls were run with a time-delay offset to facilitate the programming of the OES with the actual environmental parameters (lighting, temperature, CO_2_, RH) recorded on the ISS and seen by the plants in the orbital hardware, and volatile components of the atmosphere (e.g. ethylene and out-gassing from plastics) were equally scrubbed in both sets of hardware. The treatment of the dormant plates before launch and during the transition to the ISS and instillation into the ABRS unit was also carefully replicated in the ground controls. The conditions of the harvests (including the harvest hardware), storage of samples and the transport from the ISS to the researchers, were carefully replicated for each individual ground control.

The basic scenario for the treatment of the biological material is provided in Table 1.

**Table 1:** The operations outline both the flight and ground control experiments

**Operation**	**Flight environment/duration**	**Ground control environment/duration**
Dormant plates prepared for Turn Over to Shuttle operations	KSC laboratory (22 – 25 C)	KSC laboratory (22 – 25 C)
Plates stowed in Nomex transport bag	KSC laboratory (22 – 25 C)/2 days	KSC laboratory (22 – 25 C)/2 days
Launch to ISS and loiter on station before integration into ABRS and germination	Shuttle middeck locker then ISS EXPRESS Rack (22 – 25 C)/3–25 days	OES Shuttle middeck locker/3–25 days on at least 24 hour delay from flight
Active growth period each plate	ISS ABRS unit 22–24 C/12–14 days	OES ABRS unit 22–24 C/12–14 days on at least 24 hour delay from flight
Photography and harvest of each plate to KFT	ISS work areas (22–24 C)/1–2 hrs	OES work areas (22–24 C)/1–2 hrs on at least 24 hour delay from flight
RNAlater soak in KFT	ISS work areas (22–24 C)/12–24 hrs	OES work areas (22–24 C)/12–24 hrs on at least 24 hour delay from flight
KFT Stowage on ISS	MELFI (between −35 and −95 C)/8–32 days	cryo freezer at KSC (−80 C)/8–32 days on at least 24 hour delay from flight
Return to KSC laboratory	Double Cold Bag (−32 C)/2–3 days	cryo freezer at KSC (−80)/2–3 days
Storage at KSC	cryo freezer at KSC (−80)/2–7 days	cryo freezer at KSC (−80)/2–7 days
Transition to PIs’ laboratory	Transport on dry ice then to cryo freezer (−80)	Transport on dry ice then to cryo freezer (−80)

Each flight experiment was paired with a ground control that was run in parallel, on the ground, having been informed by environmental and operations data from the ISS on at least a 24 hour delay. In this manner, each flight operation was precisely replicated in the ground controls. Although there was variability in the durations of the dormancy period and the cold storage periods among the runs, for each individual experiment, the conditions were precisely replicated between each flight and ground control experiment pair.

### Plant lines and seed dormancy

*Arabidopsis* seeds (12 to 18 seeds per plate) were planted aseptically on the surface of 10 cm^2^ solid media plates [56]. The three GFP (Green fluorescent Protein [57]) reporter gene lines were Adh:GFP (alcohol dehydrogenase promoter [58]); DR5r:GFP (synthetic auxin response element composed of five AuxRE elements; gift of T. Guilfoyle [59]); and 35s:GFP (driven by the CaMV35s promoter [58]). Seeds and seeded plates were prepared in such a way as to maintain dormancy in light-tight coverings until the initiation of the experiment on orbit [39]. The plated seeds remained dormant until activated by exposure to light in the ABRS growth hardware, which initiated germination.

### Experiment unique equipment and environmental conditions

Both the flight and ground control plates were grown in the ABRS [60]. The ABRS provided temperature control, light control, and circulation of air that was scrubbed to remove volatile organic compounds (VOCs). On orbit, the dormant seeded plates were unwrapped from their coverings and installed in the GFP Imaging System (GIS), which was then inserted into an ABRS (Figure 1A, 1B). The GIS held the plates within the ABRS, facilitated access by the astronaut on orbit (1C), and provided regular imaging [39,61]. After growth, all plates were harvested on orbit to RNAlater-filled KSC Fixation Tubes (KFTs) (Figure 2A, 2B) and then stowed below -40°C on the ISS [1] before return. The ground control was housed in an ABRS in the Orbital Environmental Simulator (OES) chamber in the Spaceflight Life Sciences Laboratory at Kennedy Space Center.

### Imaging

The plate in position 1 of Run 3A was imaged every 6 hours. Each imaging session consisted of a white light image to evaluate general morphology [39] and three fluorescent images that were stacked into a single image for GFP analysis with Maxim-DL [62]. Quantification of the relative intensities of GFP expression in the imaged plants was conducted with ImageJ [63].

### Sample preparation for transcriptome analyses

Three separate plates of plants from Run 2B, from three different positions in the ABRS growth chamber, were used to evaluate the transcriptome of plants grown on the ISS (Flight) versus those grown in the comparable growth chamber within the Orbital Environment Simulator (Ground Control). Total RNA was isolated from plates grown in the upper tier of the GIS (plate positions 2, 4, 6; Figure 1). A representative upper-tier plate is shown in Figure 2A [images of lower tier plates can be found in [39]. RNAlater-preserved plants returned from orbit were separated into three organ types: leaves (cut from the plant at base of each petiole), hypocotyls (the region between the root/shoot junction and the start of the leaf rosette), and roots (Figure 2C and 2D). RNA from runs 2B and 1B was extracted using RNAeasy™ mini kits (QIAGEN Sciences, MD, USA) according to the manufacturer’s protocol. Residual DNA was removed by performing an on-column digestion using an RNase Free DNase (QIAGEN GmbH, Hilden, Germany). RNA from run 2A was extracted using a mirVana miRNA Isolation Kit with Acid-Phenol:Chloroform (5:1 Solution pH 4.5+/−0.2) extraction (Ambion Life Technologies). Integrity of the RNA was evaluated using the Agilent 2100 BioAnalyzer (Agilent Technologies, Santa Clara, CA, USA).

### Microarrays

RNA from five biological replicates leaves, hypocotyls, and roots from spaceflight (FLT) and ground control (GC) samples were analyzed using ten Affymetrix GeneChip® *Arabidopsis* ATH1 Genome Arrays (A-AFFY-2). The biological replicates were collected as follows (plate numbering and configuration described in Figure 1): one from Plate 2, two from Plate 4 and two from Plate 6. Each biological replicate is comprised of six to nine individual plants. All plates were upper deck plates, and received the same light levels. Flight and ground control samples were grown, harvested and extracted with identical protocols. Details of Microarray data analyses and MAS5 Statistical algorithm and Robust Multichip Analysis (RMA) applications can be found in [14,16], and in the following section. The data have been deposited in ArrayExpress under experiment accession number E-MTAB-1264.

### Statistical methods associated with the microarray analyses

The primary statistical algorithm utilized in the array analyses was MAS5.0 (Affymetrix Expression Console™ Software). MAS5.0 was first used in pre-processing to detect present and absent probe signals; probe sets scored as absent on all arrays were removed. Next MAS5.0 was used to perform additional pre-processing for background adjustment, normalization and summarization. Comparative analyses were conducted with the normalized signal intensity values. The Student’s *t*-test was performed considering a probe-by-probe comparison between each spaceflight (FLT) probe group and each ground control (GC) probe group. Three comparative analyses were performed between FLT Leaves and GC Leaves, FLT Hypocotyls and GC Hypocotyls, FLT Roots and GC Roots, respectively. The fold change (FC) was computed based on the normalized log transformed signal intensity data for each gene locus in the FLT and GC groups. P-value corrections (q-value) were generated to measure false positive rate.

### Quantitative RT-qPCR

Applied Biosystems kits and reagents (TaqMan™) were used for the quantitative RT-qPCR [64]. Ubiquitin UBQ11 (At4g05050) served as an internal control in duplex RT-qPCR reactions. Primers and probes were designed with Primer Express and supplied by Applied Biosystems. Three biological replicates were used to generate the graph provided in Figure 5. The three replicate RNA samples used for the RT-qPCR analysis of Run 2B roots were taken from the three of the five RNA samples used for the Run 2B microarrays (both flight and ground control. The quantitative analyses from the other two TAGES flight experiments (Runs 1B and 2A) were also conducted with three biological replicates of roots from each of those experiments and their respective, corresponding ground controls. The Mean dCt (Ct target – Ct UBQ11) of 3 spaceflight replicas was calculated relative to Mean dCt (Ct target- Ct UBQ11) of 3 ground control biological replicas (ddCt) (dCt FLT- dCt GC) and the fold change was calculated as 2^(−ddCt). Additional details of RT-qPCR protocol and analysis can be found in [14,16]. The list of RT-qPCR probes and primer sets is shown in Additional file 4.

## Supplementary Material

Additional file 1 **Statistically significant differential expression in response to spaceflight among the three organ types.** There are 480 genes that show statistically significant (p < 0.01) differential expression by at least 1.9-fold in at least one organ in response to spaceflight. The genes are sorted by AtG number. Click here for file

Additional file 2 **Differential expression of 7-fold or greater in response to spaceflight among organs.** Genes that show statistically significant (p < 0.01) differential expression of 7-fold or greater in response to spaceflight among organs are highlighted as bold text in the organ column in which that level of expression is displayed. Click here for file

Additional file 3 **Cellular remodelling and hormone signaling associated genes differentially expressed genes in response to spaceflight.** The hierarchical clustering of 158 genes with statistically significant (p < 0.01) differential expression in the spaceflight environment by at least 1.9-fold in at least one of the three organs, and which have an association with cell wall remodeling and cell expansion, pathogen or wounding responses, and growth hormone signal transduction. The graphic representation of gene expression patterns is annotated with the corresponding AtG number, gene name, and notes associated with that gene’s functional association. Click here for file

Additional file 4 **RT-qPCR primers and probes.** The forward and reverse primers used for RT-qPCR anaylse of DDF1, DREB2A, TCH4, JAZ7, ELIP1, and the UBQ11 control. Primers and probes were designed with Primer Express software and supplied by Applied Biosystems. Click here for file
