# Supplementary material for: Organ-specific remodeling of the Arabidopsis transcriptome in response to spaceflight
Source: BMC Plant Biol. 2013 Aug 7;13:112. doi: 10.1186/1471-2229-13-112 (PMC3750915; doi:10.1186/1471-2229-13-112)
Supplement: Additional file 1 — Statistically significant differential expression in response to spaceflight among the three organ types. There are 480 genes that show statistically significant (p < 0.01) differential expression by at least 1.9-fold in at least one organ in response to spaceflight. The genes are sorted by AtG number. [file 1471-2229-13-112-S1.pdf]

Probe ID	AtG	Gene	Short Description	Leaf	Hypc	Root
262120_at	At1g02950	ATGSTF4	glutathione-S-transferase	0.98	0.37	0.74
264323_at	At1g04180	YUC9	putative dimethylaniline monooxygenase	0.20	0.70	1.69
263652_at	At1g04330	---	unknown	0.83	2.04	0.62
264606_at	At1g04660	---	unknown	0.86	0.27	0.33
264564_at	At1g05290	---	unknown	0.34	1.16	12.04
263229_s_at	At1g05650	---	Pectin lyase-like superfamily protein; FUNCTIONS IN: polygalacturonase	0.63	0.86	0.28
260783_at	At1g06160	ORA59	OCTADECANOID-RESPONSIVE ARABIDOPSIS AP2/ERF 59; ethylene resp	1.60	2.51	1.04
261077_at	At1g07430	PP2C	protein phosphatase 2C, highly ABA-induced PP2C gene 2	0.76	0.37	1.85
264643_at	At1g08990	PGSIP5	plant glycogenin-like starch initiation protein 5	0.11	1.55	0.97
264511_at	At1g09350	AtGolS3	putative galactinol synthase similar to GB:AAD26116 from [Brassica nap	0.32	0.49	0.35
264688_at	At1g09890	---	hypothetical protein similar to LG27/30-like gene	0.59	1.19	9.99
264459_at	At1g10160	---	transposable element gene; non-LTR retrotransposon family (LINE)	0.21	0.58	0.84
264453_at	At1g10300	---	putative GTP-binding protein similar to S. cerevisiae Lpg15p	3.89	0.86	1.37
264455_at	At1g10330	---	Tetratricopeptide repeat (TPR)-like superfamily protein	1.84	1.32	0.44
262810_at	At1g11710	---	putative salt-inducible protein	0.13	1.91	0.87
255937_at	At1g12610	DDF1	DWARF AND DELAYED FLOWERING 1	3.94	4.82	24.25
259364_at	At1g13260	RAV1	transcription factor DNA-binding protein RAV1, Encodes an AP2/B3 dom	1.67	1.68	2.48
256097_at	At1g13670	---	unknown	1.93	0.54	0.54
262857_at	At1g14930	---	major latex homologue type2, Polyketide cyclase/dehydrase and lipid tr	7.46	0.95	1.28
260744_at	At1g15010	---	unknown, seems involved in defense response to fungus	2.39	3.43	4.03
260741_at	At1g15045	---	hypothetical protein contains Pfam profile: Glutamine amidotransferase	6.77	1.99	0.96
262590_at	At1g15100	RHA2A; prot	E3 ubiquitin-protein ligase RHA2A	0.81	0.55	0.42
259502_at	At1g15670	---	Galactose oxidase/kelch repeat superfamily protein	1.99	1.89	1.82
262704_at	At1g16530	ASL9	(ASYMMETRIC LEAVES 2 LIKE 9)	0.36	0.78	1.02
261033_at	At1g17380	JAZ5	jasmonate-zim-domain protein 5	1.41	1.65	3.36
261035_at	At1g17390	---	transposable element gene; similar to RNase H domain-containing prote	0.53	1.05	0.16
255891_at	At1g17870	EGY3	ETHYLENE-DEPENDENT GRAVITROPISM-DEFICIENT AND YELLOW-GREEN	1.61	2.07	1.61
255893_at	At1g17960	---	threonyl-tRNA synthetase, putative similar to threonyl-tRNA synthetase	3.16	1.99	1.34
261405_at	At1g18740	---	unknown	1.26	1.58	3.73
256017_at	At1g19180	JAZ1	JAZ1 is a nuclear-localized protein involved in jasmonate signaling	1.21	1.78	2.33
261143_at	At1g19770	ATPUP14; pu	Member of a family of proteins related to PUP1, a purine transporter.	1.06	1.64	3.32
261247_at	At1g20070	---	hypothetical protein	0.51	0.79	0.54
255884_at	At1g20310	---	hypothetical protein	1.47	3.03	1.39
259569_at	At1g20480	---	AMP-dependent synthetase and ligase family protein; phenylpropanoid	0.17	0.51	1.45
259566_at	At1g20520	---	Potential natural antisense gene, locus overlaps with AT1G20520	2.93	2.73	3.61
256083_at	At1g20730	---	hypothetical protein	0.12	1.11	0.49
256084_at	At1g20750	---	RAD3-like DNA-binding helicase protein	0.14	0.81	0.27
256094_at	At1g20780	SAUL1	SENESCENCE-ASSOCIATED E3 UBIQUITIN LIGASE 1; ubiquitin-protein liga	0.56	0.32	0.16
262803_at	At1g21000	---	PLATZ transcription factor family protein	1.73	2.07	1.42
261456_at	At1g21050	---	hypothetical protein	0.51	0.70	0.76
260856_at	At1g21910	---	TINY-like protein; encodes a member of the DREB subfamily A-5 of ERF/	1.40	1.95	2.13
255954_at	At1g22090	emb2204	(embryo defective 2204)	0.29	1.20	0.78
264202_at	At1g22810	---	TINY-like transcription factor	4.53	0.36	7.84
264774_at	At1g22890	---	unknown	1.83	1.42	5.28
265165_at	At1g23610	---	putative OBP32pep protein	0.14	0.99	1.93
265184_at	At1g23710	---	unknown	1.85	2.30	3.14
264862_at	At1g24330	---	ARM repeat superfamily protein; FUNCTIONS IN: ubiquitin-protein ligas	1.06	1.37	2.10
255733_at	At1g25400	---	unknown	2.36	1.97	2.35
245871_at	At1g26290	---	hypothetical protein	0.88	0.83	0.11
261263_at	At1g26790	---	H-protein promoter binding factor-2b	0.32	1.68	0.77
257407_at	At1g27100	---	unknown	0.85	1.37	2.25
262318_at	At1g27620	---	putative hypersensitivity-related protein; HXXXD-type acyl-transferase f	5.28	0.90	0.82
261443_at	At1g28480	GRX480	glutaredoxin, putative similar to glutaredoxin; electron carrier/ protein	1.80	2.85	3.56
261713_at	At1g32640	MYC2	Encodes a MYC-related transcriptional activator; jasmonate insensitive;	1.28	1.77	2.57
261233_at	At1g32810	---	RING/FYVE/PHD zinc finger superfamily protein	0.18	0.49	1.51
256421_at	At1g33500	---	hypothetical protein	1.40	0.15	2.20
261984_at	At1g33760	---	TINY-like protein; encodes a member of the DREB subfamily A-4 of ERF/	2.20	2.38	10.27
256026_at	At1g34170	ARF13	auxin response factor 13	0.18	0.85	2.28
262564_at	At1g34330	---	anionic peroxidase, putative similar to anionic peroxidase	1.68	0.97	0.26
259931_at	At1g34400	---	hypothetical protein	0.65	0.33	0.36
261162_at	At1g34440	---	hypothetical protein	0.49	1.89	0.89
261157_at	At1g34510	---	peroxidase ATP13a, putative	0.37	1.09	0.39

261184_at	At1g34545	---	pol polyprotein, putative; transposable element gene; copia-like retrotı	5.98	1.01	3.94
245757_at	At1g35140	PHI-1	EXL1, EXORDIUM LIKE 1, PHI-1, PHOSPHATE-INDUCED 1, located plant c	1.21	1.38	3.68
245755_at	At1g35210	---	chlorplast encoded, unk func, UV induced	1.74	2.36	4.72
246353_s_at	At1g39190	---	transposable element gene; CACTA-like transposase family (Tnp2/En/Sp	0.12	0.75	0.41
255754_at	At1g43040	---	auxin-induced protein, putative	0.81	3.66	1.71
262724_s_at	At1g43330	---	PC-MYB2, putative similar to PC-MYB2	0.12	0.93	1.09
259453_at	At1g44090	ATGA20OX5	gibberelin 20-oxidase, putative	1.66	0.62	0.12
245246_at	At1g44224	---	Encodes a ECA1 gametogenesis related family protein	0.81	1.79	5.90
261335_at	At1g44800	---	nodulin MtN21 /EamA-like transporter family protein; membrane prote	0.39	0.76	0.81
261327_at	At1g44830	---	encodes a member of the DREB subfamily A-5 of ERF/AP2 transcription	1.89	1.38	3.10
260727_at	At1g48100	---	polygalacturonase PG1, putative; Pectin lyase-like superfamily protein;	0.68	0.48	0.71
256141_at	At1g48640	---	lysine and histidine specific transporter, putative;Transmembrane amir	0.78	4.47	0.63
261690_at	At1g50090	---	branched-chain amino acid aminotransferase, putative	0.16	0.95	0.87
245749_at	At1g51090	---	proline-rich protein, putative ; Heavy metal transport/detoxification su	0.99	2.93	11.00
265133_s_at	At1g51250	---	Plant self-incompatibility protein S1 family	1.53	4.76	2.06
262225_at	At1g53840	ATPME1	encodes a pectin methylesterase; cell wall modification	1.10	1.42	2.23
262259_s_at	At1g53870	---	hypothetical protein	1.75	1.92	0.65
263008_at	At1g54470	RPP27	Encodes a Cf-like gene in Arabidopsis that confers downy mildew resista	1.64	5.43	0.92
245628_at	At1g56650	PAP1	(PRODUCTION OF ANTHOCYANIN PIGMENT 1) similar to anthocyanin2 (	0.79	0.43	0.40
246397_at	At1g58190	AtRLP9	receptor like protein 9 (RLP9) contains similarity to disease resistance p	0.98	0.35	0.25
245840_at	At1g58420	---	hypothetical protein	1.09	1.65	3.36
245842_at	At1g58430	RXF26	proline-rich protein, ; carboxylesterase/ hydrolase, acting on ester bon	6.28	0.24	0.23
262904_at	At1g59725	---	DNAJ heat shock family protein; chaperone, putative similar to chapero	1.39	5.31	1.35
264217_at	At1g60190	PUB19	Encodes PUB19, a plant U-box armadillo repeat protein. Involved in salt	2.04	2.36	3.25
259714_at	At1g60980	ATGA20OX4	putative gibberellin 20-oxidase	0.10	1.93	0.31
264753_at	At1g61490	---	S-locus lectin protein kinase family protein; receptor kinase	0.64	2.64	1.06
265033_at	At1g61520	LHCA3	PSI type III chlorophyll a/b-binding proteinthaliana];supported by full-le	0.45	0.88	0.99
264733_at	At1g62170	---	phloem serpin-1, putative; Serine protease inhibitor (SERPIN) family pro	0.81	0.25	7.06
265113_at	At1g62460	---	transposable element gene; copia-like retrotransposon family	0.41	2.30	2.89
261556_s_at	At1g63390	---	FAD/NAD(P)-binding oxidoreductase family protein	3.81	0.61	0.70
260327_at	At1g63840	---	putative RING zinc finger protein; RING/U-box superfamily protein; FUN	1.06	1.99	1.41
259793_at	At1g64380	---	member of the DREB subfamily A-6 of ERF/AP2 transcription factor	0.39	1.11	0.75
263139_at	At1g65110	---	Ubiquitin carboxyl-terminal hydrolase-related protein	6.68	1.27	1.52
257453_at	At1g65130	---	Ubiquitin carboxyl-terminal hydrolase-related protein	5.24	0.86	0.12
263141_at	At1g65210	---	Galactose-binding protein	2.11	0.22	3.63
256515_at	At1g66020	---	terpene synthase,Terpenoid cyclases/Protein prenyltransferases superf	4.86	1.00	1.10
260135_at	At1g66400	---	Encodes a calmodulin-like protein. Regulates nitric oxide levels and trar	1.39	1.39	2.57
260132_s_at	At1g66640	---	RNI-like superfamily protein	0.14	0.64	0.39
262229_at	At1g68620	---	alpha/beta-Hydrolases superfamily protein	2.81	0.88	2.08
256300_at	At1g69490	NAP	unknown protein similar to N-term half of NAC domain protein NAM	1.89	2.10	1.10
260419_at	At1g69730	---	Wall-associated kinase family protein;	0.71	0.84	2.13
264314_at	At1g70420	---	unknown protein ; supported by cDNA: gi_15010575_gb_AY045589.1_	3.51	2.07	1.56
261503_at	At1g71691	---	GDSL-like Lipase/Acylhydrolase superfamily protein	0.41	1.60	2.73
262366_at	At1g72890	---	Disease resistance protein (TIR-NBS class)	2.33	1.67	1.13
262382_at	At1g72920	---	virus resistance protein, Toll-Interleukin-Resistance (TIR) domain family	1.80	1.49	2.99
262373_at	At1g73120	---	hypothetical protein	1.65	1.69	0.31
245731_at	At1g73500	MKK9	MAP kinase, putative similar to MAP kinase kinase 5	1.30	1.44	2.16
245777_at	At1g73540	atnudt21 (Ar	nudix hydrolase 21-like; contains similarity to diphosphoinositol polypho	1.03	1.77	4.96
260227_at	At1g74450	---	unknown	1.28	1.72	5.66
261748_at	At1g76070	---	hypothetical protein	1.05	2.04	1.17
259975_at	At1g76470	---	putative cinnamoyl-CoA reductase; lignin biosynthes	0.79	0.88	2.00
259729_at	At1g77640	---	encodes a member of the DREB subfamily A-5 of ERF/AP2 transcription	1.87	1.73	3.48
259709_at	At1g77655	---	Expressed protein	1.04	3.20	2.22
259676_at	At1g77730	---	Pleckstrin homology (PH) domain superfamily protein; FUNCTIONS IN: p	0.17	1.71	0.55
260773_at	At1g78440	ATGA2OX1	gibberellin 2- oxidase (gibberellin 2-oxidase 1); gibberellin 2-beta-dioxy	0.16	0.56	0.57
264279_s_at	At1g78820	---	glycoprotein(EP1), putative; D-mannose binding lectin protein	0.75	1.54	1.95
261892_at	At1g80840	WRKY40; tra	transcription factor, putative similar to WRKY transcription factor; Path	1.71	2.81	7.46
265732_at	At2g01300	---	unknown	1.39	1.93	1.95
266174_at	At2g02440	---	hypothetical protein	0.35	0.92	1.73
263363_at	At2g03850	---	putative cold-regulated protein	4.38	1.58	1.05
266037_at	At2g05940	---	RIPK - Encodes a receptor-like cytoplasmic kinase that phosphorylates t	1.41	1.66	3.84
265552_at	At2g07560	AHA6	putative plasma membrane proton ATPase	1.13	1.48	0.17
265234_at	At2g07721	---	hypothetical protein	0.15	4.38	0.69

263815_at	At2g10020	---	hypothetical protein	0.91	0.37	0.48
263394_at	At2g10465	---	transposable element gene; similar to replication protein-related	1.30	6.15	0.41
265748_at	At2g10620	---	putative Athila retroelement ORF1 protein	0.61	3.03	11.08
265397_at	At2g11090	---	transposable element gene	0.10	1.13	1.60
263390_at	At2g11830	---	hypothetical protein similar to Ta1-1 reverse transcriptase; transposabl	0.62	0.12	4.99
263295_at	At2g14210	AGL44	(AGAMOUS-LIKE 44); DNA binding / transcription factor; putative MADS	0.18	0.74	0.50
265600_at	At2g14230	---	transposable element gene; CACTA-like transposase family (Ptta/En/Spr	0.12	1.59	0.50
257387_s_at	At2g14510	---	putative receptor-like protein kinase	0.40	0.19	0.51
265889_at	At2g15130	---	Plant basic secretory protein (BSP) family protein; defense response	0.41	8.82	1.41
265497_at	At2g15720	---	putative non-LTR retroelement reverse transcriptase	0.23	0.50	1.62
265482_at	At2g15780	---	Plastocyanin-like domain-containing protein	0.59	0.42	1.27
265536_at	At2g15880	---	Leucine-rich repeat (LRR) family protein; structural constituent of cell w	2.33	1.96	3.32
257455_s_at	At2g16100	---	hypothetical protein	0.16	1.14	0.57
263602_at	At2g16360	---	40S ribosomal protein S25	0.59	1.60	0.50
265359_at	At2g16720	MYB7	putative MYB family transcription factor (response to salt and salicylic	2.95	2.36	2.45
265408_at	At2g16820	---	Mutator-like transposase similar to MURA transposase of maize Mutat	2.36	4.99	1.96
266526_at	At2g16980	---	putative tetracycline transporter protein	0.29	2.23	1.01
263418_at	At2g17210	---	putative selenium-binding protein; Tetratricopeptide repeat (TPR)-like s	6.59	1.33	3.58
257408_at	At2g17310	SON1 (SUPP	(SUPPRESSOR OF NIM1-1 1) Encodes an F-Box protein that regulates a	0.26	1.34	2.41
264846_at	At2g17850	---	senescence-associated protein contains similarity to ketoconazole resis	0.81	1.24	0.50
266945_at	At2g18880	VEL2	((VERNALIZATION5/VIN3-LIKE)) hypothetical protein predicted by genef	0.44	0.34	5.70
265589_at	At2g20170	---	hypothetical protein	0.59	1.36	7.26
265387_at	At2g20670	---	unknown cloroplast gene	2.83	2.06	0.95
263746_at	At2g21460	---	putative retroelement pol polyprotein	1.37	0.15	2.25
263753_at	At2g21490	LEA	(DEHYDRIN LEA) putative dehydrin	0.16	0.82	1.33
264000_at	At2g22500	UCP5	(UNCOUPLING PROTEIN 5); putative mitochondrial dicarboxylate carrie	0.90	1.16	3.18
266455_at	At2g22760	---	putative bHLH transcription factor	1.29	5.90	2.03
267252_at	At2g23100	---	Cysteine/Histidine-rich C1 domain family protein	0.72	2.00	1.92
245075_at	At2g23180	CYP96A1	putative cytochrome P450	1.37	7.62	0.56
267149_s_at	At2g23500	---	Mutator-like transposase similar to MURA transposase of maize Mutat	0.34	0.16	0.52
257392_at	At2g24450	FLA3	(FASCICLIN-LIKE ARABINOGALACTAN PROTEIN 3 PRECURSOR)	0.27	2.07	0.67
264379_at	At2g25200	---	hypothetical protein	2.16	1.77	0.78
265606_s_at	At2g25550	---	putative non-LTR retroelement reverse transcriptase	0.11	0.37	2.73
266316_at	At2g27080	---	a LEA	1.06	1.54	2.95
265648_at	At2g27500	---	putative beta-1,3-glucanase ;Glycosyl hydrolase superfamily protein; F	1.62	1.61	2.01
266259_at	At2g27830	---	unknown protein	1.74	2.35	1.21
266290_at	At2g29490	ATGSTU1	putative glutathione S-transferase	2.58	1.10	0.74
266834_s_at	At2g30020	---	putative protein phosphatase 2C; Encodes AP2C1. Belongs to the clade	1.68	2.19	4.00
263478_at	At2g31880	---	putative receptor-like protein kinase ; Encodes a putative leucine rich r	1.01	1.67	2.51
263475_at	At2g31945	---	Expressed protein	3.94	3.12	1.22
267056_at	At2g32470	---	F-box associated ubiquitination effector family protein	1.16	0.96	3.92
267644_s_at	At2g32880	---	TRAF-like family protein	0.84	1.51	9.32
255844_at	At2g33580	---	Protein kinase superfamily protein; FUNCTIONS IN: kinase activity; INVC	1.41	1.87	3.73
267022_at	At2g34230	---	hypothetical protein	0.62	0.61	4.86
267043_at	At2g34330	---	unknown protein	0.32	2.79	0.41
267047_at	At2g34370	---	putative selenium-binding protein	0.39	1.37	1.84
266901_at	At2g34600	JAZ7	(JASMONATE-ZIM-DOMAIN PROTEIN 7); protein TIFY 5B (responds to ch	3.32	6.11	2.51
267427_at	At2g34830	WRKY35 (WI	putative WRKY-type DNA binding protein; embryo development ending	0.86	1.93	0.55
266545_at	At2g35290	---	hypothetical protein	1.41	1.84	5.78
265842_at	At2g35700	ERF38	ethylene-responsive transcription factor ; putative AP2 domain transcri	1.27	0.37	1.29
265802_at	At2g35733	---	predicted protein	1.31	2.28	1.16
263935_at	At2g35930	PUB23	(PLANT U-BOX 23); U-box domain containing E3 ubiquitin ligase that is i	2.28	1.93	3.18
263953_at	At2g36050	OFP15	(ARABIDOPSIS THALIANA OVATE FAMILY PROTEIN 15)	1.78	1.02	3.46
263931_at	At2g36220	---	unknown protein	1.18	1.56	2.50
265211_at	At2g36640	ATECP63	late embryogenesis abundant protein (AtECP63) ; (EMBRYONIC CELL PF	0.85	1.72	12.73
265204_at	At2g36650	---	hypothetical protein predicted by genscan	0.52	1.79	2.83
265200_s_at	At2g36790	UGT73C6	UDP-glucosyl transferase 73C6 -putative	2.17	1.21	2.00
263890_at	At2g37030	---	SAUR-like auxin-responsive protein family	0.73	1.04	5.21
265471_at	At2g37130	---	putative peroxidase ATP2a	1.11	0.47	0.76
267143_at	At2g38100	---	putative peptide/amino acid transporter	0.58	0.22	0.34
266421_at	At2g38540	LP1	calmodulin binding; putative nonspecific lipid-transfer protein	0.44	1.55	0.64
267623_at	At2g39650	---	unknown protein	1.46	1.65	2.31
245086_at	At2g39820	---	putative translation initiation factor	1.09	6.73	0.69

267361_at	At2g39920	---	HAD superfamily, subfamily IIIB acid phosphatase	0.75	0.89	10.93
267357_at	At2g40000	HSPRO2	HS1 PRO-1 2-like protein (defense response to bacterium)	1.39	2.53	3.73
266052_at	At2g40740	WRKY55; tra	putative WRKY-type III DNA binding protein	0.87	0.86	6.19
257382_at	At2g40750	WRKY54; tra	member of WRKY Transcription Factor; Group III	1.00	1.45	2.17
245119_at	At2g41640	---	Glycosyltransferase family 61	2.14	2.33	4.59
265877_at	At2g42380	bZIP transcri	unknown protein ; supported by cDNA: gi_15100052_gb_AF401299.1_	1.19	2.33	6.77
265856_at	At2g42430	LBD16	(LATERAL ORGAN BOUNDARIES-DOMAIN 16) LOB-domain protein gene	0.45	0.21	0.88
263972_at	At2g42760	---	unknown protein	1.14	2.11	2.68
265264_at	At2g42930	---	Carbohydrate-binding X8 domain superfamily protein	0.19	1.31	1.32
266446_at	At2g43310	---	Ribosomal L18p/L5e family protein	0.78	0.42	0.57
260558_at	At2g43600	---	putative endochitinase	0.12	0.92	1.74
260556_at	At2g43620	---	putative endochitinase	0.90	0.39	1.28
260607_at	At2g43700	---	Concanavalin A-like lectin protein kinase family	0.43	0.68	0.30
260559_at	At2g43860	---	putative polygalacturonase; Pectin lyase-like superfamily protein	0.44	0.65	1.23
267230_at	At2g44080	ARL (ARGOS	Encodes ARL, a gene similar to ARGOS involved in cell expansion-depen	1.82	2.10	1.73
267384_at	At2g44370	---	cysteine/histidine-rich C1 domain-containing protein	0.95	0.44	1.01
266883_at	At2g44810	DAD1	(DEFECTIVE ANTHER DEHISCENCE 1); phospholipase A1/ triacylglycerol l	0.44	0.54	0.25
266124_at	At2g45080	cycp3;1	(cyclin p3;1); cyclin-dependent protein kinase; putative PREG1-like nega	1.72	0.94	2.14
266597_at	At2g46130	WRKY43; tra	putative WRKY-type DNA binding protein ;supported by cDNA: gi:15384	0.39	0.84	0.53
266753_at	At2g46990	IAA20	auxin-induced protein (IAA20) (INDOLE-3-ACETIC ACID INDUCIBLE 20); t	1.68	0.14	1.49
266752_at	At2g47000	ABCB4	(ATP BINDING CASSETTE SUBFAMILY B4) auxin polar transport	2.17	0.90	1.02
260581_at	At2g47190	MYB2	MYB transcription factor (Atmyb2)(MYB DOMAIN PROTEIN 2); DNA binc	0.85	1.27	3.27
245171_at	At2g47560	---	RING/U-box superfamily protein	1.39	1.21	0.50
266486_at	At2g47950	---	unknown	1.57	2.30	2.73
**265772_at**	At2g48010	RKF3	putative protein kinase; (RECEPTOR-LIKE KINASE IN IN FLOWERS 3); kin	1.48	1.55	1.99
258856_at	At3g02040	SRG3	(senescence-related gene 3); glycerophosphodiester phosphodiesteras	2.55	1.41	1.29
258629_at	At3g02850	SKOR	stelar K+ outward rectifying channel (SKOR) identical to stelar K+ outwa	0.22	1.25	0.82
259044_at	At3g03430	---	pollen allergen Bra r II Calcium-binding EF-hand	7.84	2.04	0.93
259331_at	At3g03840	---	putative auxin-induced protein similar to SAUR	0.76	1.40	0.11
258583_at	At3g04100	AGL57	AGAMOUS-like 57 ; putative SRF-type transcription factor (ATAF1) belor	0.54	4.99	1.17
258792_at	At3g04640	---	hypothetical protein	1.06	1.89	2.83
259015_at	At3g07350	---	unknown protein	2.17	2.14	1.61
259246_s_at	At3g07730	---	hypothetical protein	2.83	1.22	3.51
258671_at	At3g08560	VHA-E2	(VACUOLAR H+-ATPASE SUBUNIT E ISOFORM 2); putative vacuolar ATP	2.55	1.11	1.03
258682_at	At3g08720	S6K2	(ARABIDOPSIS THALIANA SERINE/THREONINE PROTEIN KINASE 2); kina	1.57	1.48	1.95
259206_at	At3g09040	---	hypothetical protein	2.07	1.00	5.82
259026_at	At3g09240	---	putative protein kinase similar to hypothetical proteins GB:AAC13615,	0.37	1.27	0.66
259035_at	At3g09430	---	hypothetical protein	0.87	0.19	1.92
258753_at	At3g09530	ATEXO70H3	(exocyst subunit EXO70 family protein H3); protein binding	1.16	2.51	4.53
258650_at	At3g09830	---	putative protein kinase similar to protein kinase (APK1A)	1.03	1.21	2.14
258940_at	At3g09930	---	putative lipase acylhydrolase	0.21	0.28	0.52
259144_at	At3g10180	---	putative kinesin-like centromere protein	0.81	1.85	0.12
258959_at	At3g10600	CAT7	(CATIONIC AMINO ACID TRANSPORTER 7); cationic amino acid transme	0.81	0.62	3.39
258779_at	At3g11870	---	putative protein kinase	0.29	3.66	0.31
256289_s_at	At3g12230	scpl14	serine carboxypeptidase, (serine carboxypeptidase-like 14); serine-type	0.10	1.88	1.47
257603_at	At3g13820	---	F-box and associated interaction domains-containing protein	0.28	0.33	1.29
258395_at	At3g15500	ANAC055	putative jasmonic acid regulatory protein; Encodes an ATAF-like NAC-dc	2.62	1.64	0.67
259375_at	At3g16370	---	putative APG protein similar to anter-specific proline-rich protein APG p	0.57	1.89	0.91
258438_at	At3g17230	---	invertase/pectin methylesterase inhibitor family protein;	9.19	0.86	3.29
258349_at	At3g17609	HYH	Encodes a homolog of HY5 (HYH). Involved in phyB signaling pathway	1.27	0.46	0.57
257550_at	At3g18460	---	hypothetical protein	0.17	1.52	0.97
257747_at	At3g18700	---	hypothetical protein	1.95	1.84	0.20
257802_at	At3g18770	---	Autophagy-related protein 13; LOCATED IN: chloroplast	1.44	1.39	0.50
258016_at	At3g19350	MPC	(MATERNALLY EXPRESSED PAB C-TERMINAL); poly(A) binding	0.83	0.74	0.46
257076_at	At3g19680	---	unknown protein	1.01	1.40	2.41
257976_at	At3g20840	PLT1	(PLETHORA 1); transcription factor;putative transcription factor contai	1.49	0.15	1.40
257950_at	At3g21780	UGT71B6	(UDP-glucosyl transferase 71B6 / ABA glucosyltransferase	7.78	2.93	1.32
256938_at	At3g22500	ATECP31	LEA proteingi_15450430_gb_AY052316.1_	4.11	1.19	1.82
258321_at	At3g22840	ELIP1	early light-induced protein (EARLY LIGHT-INDUCABLE PROTEIN); chloro	0.67	0.84	0.50
257919_at	At3g23250	MYB15	myb-related transcription factor, (MYB DOMAIN PROTEIN 15); DNA bir	2.77	1.93	5.86
257161_at	At3g24280	SMAP2	(small acidic protein 2)	0.15	1.29	0.86
256898_at	At3g24650	ABI3	(ABA INSENSITIVE 3); DNA binding / basal transcription repressor/ trans	0.67	0.36	0.60
256760_at	At3g25650	ASK15	SKP1/ASK1 (At15), (ARABIDOPSIS SKP1-LIKE 15); protein binding / ubiq	0.45	4.76	2.77

257644_at	At3g25780	AOC3	(ALLENE OXIDE CYCLASE 3); allene-oxide cyclase	1.17	1.46	2.75
257012_at	At3g26120	TEL1	RNA-binding protein, (TERMINAL EAR1-LIKE 1); RNA binding / nucleic ac	2.64	0.49	1.69
257624_at	At3g26220	CYP71B3	cytochrome P450 monooxygenase (CYP71B3) ; electron carrier/ heme b	3.46	0.99	0.95
257625_at	At3g26230	CYP71B24	cytochrome P450, putative contains Pfam profile: PF00067 cytochrome	1.93	1.85	1.11
256874_at	At3g26320	CYP71B36	cytochrome P450, putative	2.07	0.71	0.17
258279_at	At3g26870	---	putative self-incompatibility protein	0.55	0.19	2.13
257147_at	At3g27270	---	TRAM, LAG1 and CLN8 (TLC) lipid-sensing domain containing protein	0.49	0.86	0.64
258232_at	At3g27750	---	EMBRYO DEFECTIVE 3123	0.86	1.18	0.25
257308_at	At3g28120	---	unknown protein	0.39	4.82	0.78
257071_at	At3g28180	ATCSLC04 (C	(CELLULOSE-SYNTHASE LIKE C4); cellulose synthase/ transferase, transf	1.05	1.59	1.95
256633_at	At3g28340	GATL10	(Galacturonosyltransferase-like 10); polygalacturonate 4-alpha-galactur	1.21	1.71	4.03
257842_at	At3g28390	PGP18	P-glycoprotein, putative (P-GLYCOPROTEIN 18); ATPase, coupled to tra	0.14	0.45	3.94
258003_at	At3g29030	EXPA5	expansin At-EXP5 identical to expansin At-EXP5 (EXPANSIN A5)	0.49	0.76	1.69
245223_at	At3g29791	---	unknown protein	0.16	1.21	2.23
257307_at	At3g30210	MYB121	myb-like transcription factor, putative (MYB DOMAIN PROTEIN 121); D	0.63	0.23	0.84
256705_at	At3g30290	CYP702A8	cytochrome P450, putative similar to GB:C71417 from [Arabidopsis tha	0.64	6.15	1.71
256708_at	At3g30320	---	hypothetical protein	1.73	0.90	0.11
256571_at	At3g30730	---	hypothetical protein	0.09	1.13	0.97
256003_at	At3g31430	---	Ta11 non-LTR retroelement protein	0.75	0.20	0.73
252848_at	At3g42190	---	transposable element gene; similar to cysteine-type peptidase	0.72	1.80	0.22
252802_at	At3g42350	---	transposable element gene	6.45	1.68	0.54
252818_at	At3g42590	---	transposable element gene	1.16	0.64	4.11
252747_at	At3g43320	---	putative protein retrotransposon finger protein	0.23	3.71	1.02
252753_at	At3g43500	---	hypothetical protein	0.43	0.31	0.16
252702_at	At3g43730	---	transposable element gene	1.40	0.25	0.71
252705_at	At3g43760	---	transposable element gene	0.15	0.48	0.90
252679_at	At3g44260	---	CCR4-associated factor 1-like protein CAF1_MOUSE CCR4-ASSOCIATED	1.20	1.67	4.96
252661_at	At3g44450	---	putative protein	0.53	0.78	0.71
252657_at	At3g44780	---	Cysteine proteinases superfamily protein	0.13	1.27	0.70
252592_at	At3g45640	NAXT1	mitogen-activated protein kinase 3 ;(NITRATE EXCRETION TRANSPORTE	1.18	1.44	1.92
252544_at	At3g45810	---	respiratory burst oxidase - like protein (RbohD) respiratory burst oxidas	0.15	0.43	1.09
252549_at	At3g45860	---	protein kinase - like receptor-like protein kinase RLK3, Arabidopsis thali	1.92	0.75	1.84
252553_at	At3g45910	---	putative protein	0.29	1.62	1.73
252563_at	At3g45970	ATEXLA1	(ARABIDOPSIS THALIANA EXPANSIN-LIKE A1) putative protein cim1 indu	1.16	1.82	2.11
252474_at	At3g46620	---	zinc finger (C3HC4-type RING finger) family protein	1.09	1.74	2.85
252460_at	At3g47230	---	transposable element gene	0.32	7.11	3.73
252421_at	At3g47540	---	endochitinase-like protein BASIC ENDOCHITINASE CHB4 PRECURSOR - c	0.88	1.89	1.14
252314_at	At3g49400	---	Transducin/WD40 repeat-like superfamily protein	1.89	3.07	0.62
252258_at	At3g49450	---	F-box and associated interaction domains-containing protein	0.47	0.19	0.42
252278_at	At3g49530	anac062	NAC2-like protein NAC2 - (Arabidopsis NAC domain containing protein	1.19	1.77	2.60
252193_at	At3g50060	MYB77	R2R3-MYB transcription factor ; assoc w lateral root development	1.19	1.53	3.56
252131_at	At3g50930	BCS1	BCS1 protein-like protein Homo sapiens h-bcs1 (BCS1) mRNA, (CYTOCH	1.39	1.60	3.36
252110_at	At3g51560	---	disease resistance-like protein TMV resistance protein N, Nicotiana glut	0.63	0.42	1.89
252076_at	At3g51660	---	LS1-like protein AT-LS1 product - Tautomerase/MIF superfamily protein	0.51	0.79	0.72
252085_s_at	At3g52000	scpl37	serine-type carboxypeptidase like protein serine-type carboxypeptidase	2.83	1.38	1.54
252028_at	At3g52640	---	Zn-dependent exopeptidases superfamily protein	0.65	0.17	1.28
251925_at	At3g54000	---	hypothetical protein	1.07	1.36	2.14
251842_at	At3g54580	---	extensin precursor -like protein extensin precursor,EXT10, root specific	0.30	0.85	0.42
251829_at	At3g55020	---	Ypt/Rab-GAP domain of gyp1p superfamily protein	0.69	1.02	0.36
251824_at	At3g55090	---	ABC transporter - like protein ATP-binding cassette-sub-family G-memb	0.14	0.47	2.58
251836_at	At3g55190	---	lipase -like protein monoglyceride lipase, Mus musculus	2.16	4.17	0.66
251727_at	At3g56290	---	putative protein	0.62	0.46	0.61
251705_at	At3g56400	WRKY70; tra	member of WRKY Transcription Factor; Group III. Function as activator c	1.09	1.73	2.25
246293_at	At3g56710	SIB1	Sig1 (sigma factor)binding protein; SibI is imported into chloroplasts an	1.22	1.60	2.45
251683_at	At3g57120	---	Protein kinase superfamily protein	2.17	1.19	1.18
251609_at	At3g57900	---	putative protein	1.28	1.51	5.54
251570_at	At3g58300	---	Arabidopsis phospholipase-like protein (PEARLI 4) family	0.16	1.03	0.33
251521_at	At3g59420	ACR4	(ARABIDOPSIS CRINKLY4); kinase/ transmembrane receptor protein kin	0.82	0.80	1.99
251443_at	At3g59940	---	Galactose oxidase/kelch repeat superfamily protein	1.74	2.00	1.56
251336_at	At3g61190	BAP1	(BON ASSOCIATION PROTEIN 1); phospholipid binding / protein binding	1.85	2.81	4.03
251335_at	At3g61400	---	1-aminocyclopropane-1-carboxylate oxidase-like protein 1-aminocyclop	0.40	3.58	0.65
251278_at	At3g61780	emb1703	putative protein hypothetical protein - Arabidopsis thaliana, PIR:F7142	1.06	0.90	1.96
251259_at	At3g62260	---	(embryo defective 1703)	1.33	1.82	3.46

251228_at	At3g62710	---	beta-D-glucan exohydrolase-like protein exhydrolase II - Zea mays, EMI	0.24	0.82	1.04
251192_at	At3g62720	XT1	alpha galactosyltransferase-like protein alpha galactosyltransferase - (X	1.05	1.62	2.69
255632_at	At4g00680	ADF8	putative actin-depolymerizing factor ; (ACTIN DEPOLYMERIZING FACTO	5.17	4.99	0.61
255400_at	At4g03760	---	hypothetical protein	3.56	0.22	2.30
255347_at	At4g03810	---	putative retrotransposon protein	0.14	0.58	1.17
255349_at	At4g03830	---	hypothetical protein	0.66	2.91	6.06
255372_at	At4g04120	---	putative reverse transcriptase	1.56	1.99	6.50
255245_at	At4g05630	---	hypothetical protein	0.96	1.14	0.21
255191_at	At4g07370	---	transposable element gene; gypsy-like retrotransposon family (Athila),	0.74	5.24	2.20
255172_x_at	At4g08000	---	putative transposon protein similar to A. majus TNP2	0.30	0.52	0.18
255141_at	At4g08420	---	transposable element gene; Mutator-like transposase family	0.54	0.72	7.73
255111_at	At4g08780	---	peroxidase C2 precursor like protein peroxidase (EC 1.11.1.7) C2 precur	0.53	0.86	0.44
255064_at	At4g08950	EXO	(EXORDIUM) putative phi-1-like phosphate-induced protein ;exordium;	0.85	1.30	2.60
255037_at	At4g09460	ATMYB6	myb6 DNA-binding protein (response to gibberellin stimulus, response t	1.54	2.01	1.09
255024_at	At4g09860	---	hypothetical protein	8.17	1.37	0.63
254954_at	At4g10910	---	hypothetical protein	2.68	2.28	1.91
254945_at	At4g10940	---	putative RING finger protein ring finger protein, Brevicoryne brassicae,	0.25	1.89	0.44
254926_at	At4g11280	ACS6	ACC synthase (AtACS-6)	1.04	1.95	3.20
254828_at	At4g12550	AIR1	putative cell wall-plasma membrane disconnecting CLCT protein (AIR1A	1.04	0.36	0.41
254733_at	At4g13760	---	putative polygalacturonase polygalacturonase, Pectin lyase-like superfa	0.12	0.55	0.47
254735_at	At4g13810	AtRLP47	putative disease resistance protein Cf-4 ,Lycopersicon hirsutum, (Recep	1.00	0.55	8.69
245624_at	At4g14090	---	glucosyltransferase like protein	0.57	0.38	0.57
245329_at	At4g14365	---	XB3 ortholog 4 in Arabidopsis thaliana (XBAT34) zinc ion binding	0.97	1.37	2.22
245568_at	At4g14650	---	hypothetical protein	0.72	2.13	0.86
245300_at	At4g16350	CBL6	(CALCINEURIN B-LIKE PROTEIN 6); calcium ion binding	1.40	0.72	0.52
245497_at	At4g16460	---	hypothetical protein	0.71	0.86	4.56
245346_at	At4g17090	CT-BMY	putative beta-amylase ; (CHLOROPLAST BETA-AMYLASE); beta-amylase	0.60	1.06	3.63
254700_at	At4g18000	---	hypothetical protein	0.15	0.86	0.61
254592_at	At4g18880	AT-HSFA4A	heat shock transcription factor - like protein heat shock transcription fa	1.28	1.53	2.13
254497_at	At4g20080	---	Phosphoribosylanthranilate transferase phosphoribosylanthranilate tra	0.81	0.55	5.66
254511_at	At4g20220	---	Reverse transcriptase	0.16	1.40	0.41
254430_at	At4g20820	---	reticuline oxidase - like protein reticuline oxidase (EC 1.5.3.9) precursor	1.27	6.50	0.86
254410_at	At4g21410	---	CRK29; serine/threonine kinase - like protein	1.93	1.02	0.97
254411_at	At4g21420	---	transposable element gene; gypsy-like retrotransposon family	3.16	0.76	0.96
254331_s_at	At4g22710	CYP706A2	cytochrome P450 - like protein flavonoid 3',5'-hydroxylase Hf1, Petunia	0.88	1.35	2.58
254240_at	At4g23496	SP1L5	(SPIRAL1-LIKE5) Regulates cortical microtubule organization. Mutant pla	0.78	0.91	0.53
254119_at	At4g24780	---	putative pectate lyase pectate lyase, Musa acuminata,	0.53	0.78	0.68
254059_at	At4g25200	ATHSP23.6-M	Arabidopsis mitochondrion-localized small heat shock protein (AtHSP23	1.09	3.27	1.44
254042_at	At4g25810	XTR6	xyloglucan endo-1,4-beta-D-glucanase (XTR-6) (XYLOGLUCAN ENDOTR	1.67	2.28	3.89
253962_at	At4g26460	---	S-adenosyl-L-methionine-dependent methyltransferases superfamily pr	1.36	1.21	0.24
253910_at	At4g27290	---	putative receptor like kinase receptor-like kinase SFR2, Brassica olerace	7.01	1.27	0.57
253839_at	At4g27890	---	HSP20-like chaperones	0.77	0.84	0.46
253853_at	At4g28130	---	putative diacylglycerol kinase (fragment) putative diacylglycerol kinase	1.52	6.73	0.59
253815_at	At4g28250	ATEXPB3 (AT	putative beta-expansin/putative beta-expansin/allergen protein; plant-	0.52	0.74	0.82
253713_at	At4g29370	---	Galactose oxidase/kelch repeat superfamily protein; CONTAINS InterPro	1.22	0.21	2.77
253627_at	At4g30650	---	low temperature and salt responsive protein homolog low temperature	0.45	1.01	0.76
253604_at	At4g30970	---	hypothetical protein	1.45	0.15	0.22
253485_at	At4g31800	WRKY18; tra	Pathogen-induced transcription factor	1.39	2.43	3.03
253504_at	At4g31960	---	hypothetical protein	1.36	3.92	0.61
253472_at	At4g32230	---	hypothetical protein	0.18	0.60	0.34
253405_at	At4g32800	---	transcription factor TINY homolog encodes a member of the DREB subf	1.18	0.90	2.00
253416_at	At4g33070	PDC1	pyruvate decarboxylase-1 (Pdc1)	1.01	0.72	0.42
253356_at	At4g33390	---	putative protein myosin II heavy chain - Naegleria fowleri,PID:g135376	0.14	0.44	0.47
253298_at	At4g33560	---	putative protein ;supported by full-length cDNA: Ceres:17194.	1.01	1.13	0.52
253323_at	At4g33920	---	Protein phosphatase 2C family protein	1.13	1.44	2.35
253292_at	At4g33985	---	Expressed protein	0.94	1.39	2.41
253284_at	At4g34150	---	Calcium-dependent lipid-binding (CaLB domain) family protein	0.88	1.52	2.41
253244_at	At4g34580	COW1	putative protein SEC14 protein, Saccharomyces cerevisiae, (CAN OF W	0.96	0.76	0.45
253209_at	At4g34830	---	putative protein membrane-associated salt-inducible protein, Member	2.99	0.30	0.50
253219_at	At4g34990	AtMYB32 (m	MYB-like protein myb-related protein Y49, Arabidopsis thaliana, PIR:S5	1.33	2.00	2.10
253140_at	At4g35480	RHA3B	RING-H2 finger protein RHA3b ;supported by full-length cDNA: Ceres:3	1.17	1.45	2.07
252984_at	At4g37990	ELI3-2	cinnamyl-alcohol dehydrogenase ELI3-2 ; (ELICITOR-ACTIVATED GENE 3	3.36	1.59	1.51
252958_at	At4g38620	MYB4; DNA	putative transcription factor (MYB4) ; supported by cDNA: gi_3941411_	2.64	2.10	1.40

252882_at	At4g39675	---	Expressed protein	0.86	2.25	0.41
251103_at	At5g01700	---	putative protein protein phosphatase type 2C - Saccharomyces cerevisi	2.27	1.00	1.58
251064_at	At5g01860	---	putative zinc finger protein several zinc finger proteins	0.22	0.80	0.42
250905_at	At5g03640	---	protein kinase -like protein protein kinase 5, Arabidopsis thaliana, PIR:J	0.43	5.31	0.68
245711_at	At5g04340	ZAT6	putative c2h2 zinc finger transcription factor	2.20	3.34	2.91
250781_at	At5g05410	DREB2A; DN	DREB2A (dbj|BAA33794.1) ; supported by cDNA: gi_3738229_dbj_AB0(	1.83	4.63	3.61
250561_at	At5g08030	---	glycerophosphodiester phosphodiesterase - like	9.65	0.67	1.00
246018_at	At5g10695	---	Expressed protein	1.36	2.11	3.68
250396_at	At5g10970	---	zinc finger -like protein CCHH finger protein 3, Arabidopsis thaliana, PIR	0.26	0.86	0.73
245906_at	At5g11070	---	putative protein	0.97	1.71	3.27
250171_at	At5g14300	ATPHB5	prohibitin - like protein prohibitin, Arabidopsis thaliana, EMBL:ATU691!	0.56	0.19	1.54
250091_at	At5g17340	---	unknown protein	5.58	0.57	1.15
246426_at	At5g17430	BBM	(BABY BOOM); DNA binding / transcription factor ovule development	2.19	3.58	0.62
250053_at	At5g17850	---	potassium-dependent sodium-calcium exchanger - like protein cone so	1.21	2.57	3.18
250071_at	At5g18000	---	Encodes VERDANDI (VDD), a putative transcription factor belonging to t	0.78	1.53	8.69
246071_at	At5g20150	SPX1	ids4-like protein ids-4 protein - Hordeum vulgare, (SPX DOMAIN GENE :	2.57	1.26	1.25
246118_at	At5g20340	BG5	beta-1,3-glucanase bg5	0.56	0.59	0.21
245970_at	At5g20710	BGAL7	beta-galactosidase ; (beta-galactosidase 7); beta-galactosidase	2.83	0.17	0.86
249928_at	At5g22250	---	CAF1B CCR4-associated factor-like protein	1.56	1.84	3.58
249939_at	At5g22430	---	Pollen Ole e 1 allergen and extensin family protein	0.28	0.31	0.40
249896_at	At5g22530	---	unknown protein	0.69	1.79	2.11
249746_at	At5g24590	TIP	NAC2-like protein (TCV-INTERACTING PROTEIN); transcription coactivat	1.10	1.21	2.39
246871_at	At5g26070	---	extensin - like protein pAP8 protein, Nicotiana tabacum, PIR:S33159	1.12	1.07	0.17
246872_at	At5g26080	---	extensin - like protein hydroxyproline-rich glycoprotein precursor, Nico	5.62	1.68	0.28
246854_at	At5g26200	---	mitochondrial carrier - like protein mitochondrial carrier protein, Ribes	1.40	0.77	0.42
246821_at	At5g26920	CBP60G	calmodulin-binding - like protein calmodulin-binding protein TCB60, Nic	1.41	0.86	2.07
246777_at	At5g27420	---	RING-H2 zinc finger protein-like RING-H2 zinc finger protein ATL6 - Aral	2.00	2.19	2.55
245858_at	At5g28280	---	CER1-like protein CER1-like, A.thaliana, ATCER1L19	2.48	1.67	0.17
245919_at	At5g28780	---	PIF1 helicase	0.42	1.15	0.33
246669_at	At5g29958	---	galactinol synthase, putative	1.05	1.59	0.13
246102_at	At5g32590	---	myosin heavy chain-related	4.63	1.06	2.10
260184_s_at	At5g34950	---	transposable element gene	1.51	0.35	2.77
246656_at	At5g35240	---	transposable element gene	0.47	2.73	10.93
249699_at	At5g35540	---	putative protein	0.20	1.24	3.61
249705_at	At5g35580	---	serine/threonine protein kinase-like	1.41	2.01	3.51
249719_at	At5g35735	---	Auxin-responsive family protein	1.10	1.93	3.16
249673_at	At5g35920	CYP79A4P	cytochrome P450-like	0.52	1.88	0.11
249675_at	At5g35940	---	putative protein myrosinase-binding protein-like; also similar to jasmon	0.40	0.30	1.77
249662_s_at	At5g36770	---	putative protein hypothetical proteins - Arabidopsis thaliana	0.79	0.26	2.41
249664_at	At5g36810	---	putative protein histone stem-loop binding protein - Xenopus laevis, EN	3.18	1.16	10.56
249604_x_at	At5g37230	---	putative protein RING-H2 finger protein RHA3b, Arabidopsis thaliana, P	0.85	0.18	1.30
249607_at	At5g37280	---	putative protein RING-H2 finger protein RHA1b	9.92	0.90	2.17
249583_at	At5g37770	TCH2	CALMODULIN-RELATED PROTEIN 2, TOUCH-INDUCED (TCH2) ;(TOUCH 2	0.88	1.18	2.07
249520_at	At5g38670	---	Galactose oxidase/kelch repeat superfamily protein	4.66	1.18	1.80
249522_at	At5g38700	---	putative protein	0.99	1.28	2.39
249393_at	At5g40170	AtRLP54	disease resistance - like protein resistance gene Cf-4, Lycopersicon hirsu	1.54	2.51	1.84
249337_at	At5g41080	---	putative protein contains similarity to (Receptor Like Protein 54); kinase	2.39	1.39	1.48
249264_s_at	At5g41740	---	disease resistance protein-like	2.22	1.02	2.99
249234_at	At5g42200	---	RING/U-box superfamily protein	1.39	1.93	1.85
249197_at	At5g42380	CML37	(CALMODULIN LIKE 37); calcium ion binding	2.38	2.16	6.87
249205_at	At5g42600	MRN1	cycloartenol synthase(MARNERAL SYNTHASE); catalytic/ marneral synth	0.87	0.30	0.96
249208_at	At5g42650	AOS	allene oxide synthase (emb CAA73184.1) ; (ALLENE OXIDE SYNTHASE); a	1.06	1.37	2.35
249141_at	At5g43200	---	Zinc finger, C3HC4 type (RING finger) family protein	0.81	0.12	1.15
249155_at	At5g43480	---	unknown protein	0.34	0.31	0.51
249042_at	At5g44350	---	ethylene-regulated nuclear protein ERT2-like protein	0.99	1.29	2.06
248990_at	At5g45210	---	disease resistance protein-like	0.22	0.14	0.90
248993_at	At5g45240	---	putative protein contains similarity to disease resistance protein	1.55	4.86	1.13
248964_at	At5g45340	CYP707A3; (	cytochrome P450	1.39	2.58	3.86
248911_at	At5g45830	DOG1	tumor-related protein-like (DELAY OF GERMINATION 1)	4.56	0.33	0.92
248930_at	At5g46010	---	Homeodomain-like superfamily protein	0.14	0.81	1.97
248880_at	At5g46200	---	putative protein contains similarity to carboxyl-terminal proteinase	0.19	0.62	1.35
248892_at	At5g46300	---	unknown protein	1.00	6.77	0.82
248862_at	At5g46730	---	glycine-rich protein (putative C2H2 transcription factor)	0.73	0.34	0.94

248821_at	At5g47070	---	protein serine threonine kinase-like	1.31	2.04	3.34
248794_at	At5g47220	ERF2	ethylene responsive element binding factor 2 (ATERF2) (ETHYLENE RESF	1.79	1.68	3.71
248626_at	At5g48940	---	receptor protein kinase-like protein	4.66	0.77	1.01
248645_at	At5g49150	GEX2	(GAMETE EXPRESSED 2)	1.09	0.51	7.52
248611_at	At5g49520	WRKY48; tra	WRKY48 is a stress- and pathogen-induced transcriptional activator	1.39	1.38	2.62
248514_s_at	At5g50510	---	unknown protein	0.80	0.47	0.13
248490_at	At5g50940	---	RNA-binding KH domain-containing protein	3.01	1.15	1.44
248372_at	At5g51850	---	putative protein	0.54	1.22	2.07
248350_at	At5g52160	---	Bifunctional inhibitor/lipid-transfer protein/seed storage 2S albumin su	0.46	3.61	0.29
248277_at	At5g52860	---	ABC transporter-like protein	0.57	0.79	0.47
248185_at	At5g54060	UF3GT	flavonol 3-O-glucosyltransferase-like	0.54	0.33	0.56
248164_at	At5g54490	PBP1	(PINOID-BINDING PROTEIN 1); calcium ion binding / protein binding	1.62	2.71	3.61
248110_at	At5g55320	---	wax synthase-like protein	1.22	4.29	2.23
248064_at	At5g55570	---	unknown protein	1.33	0.19	0.54
248047_at	At5g56070	---	putative protein similar to unknown protein (pir||T05055)	0.55	5.43	0.92
247903_at	At5g57340	---	unknown protein ; supported by cDNA: gi_16226274_gb_AF428289.1_	1.54	2.13	1.54
247925_at	At5g57560	TCH4	TCH4 protein (gb|AAA92363.1) ; supported by cDNA: gi_14194112_gb_	0.85	1.72	5.10
247848_at	At5g58120	---	resistance protein - like disease resistance protein RPP1-WsA, Arabidop	1.21	1.09	2.77
247811_at	At5g58430	ATEXO70B1	leucine zipper-containing protein leucine zipper-containing protein, Lyc	1.07	1.67	2.33
247789_at	At5g58680	---	ARM repeat superfamily protein	1.83	8.57	15.56
247798_at	At5g58830	---	subtilisin-like serine protease contains similarity to prepro-cucumisin GI	0.23	3.29	0.79
247714_at	At5g59340	WOX2	wuschel protein - like wuschel protein, Arabidopsis thaliana, (WUSCHEL	0.50	1.12	0.49
247729_at	At5g59530	---	1-aminocyclopropane-1-carboxylate oxidase - like protein 1-aminocyclo	0.15	0.43	0.97
247655_at	At5g59820	RHL41	zinc finger protein Zat12 ;supported by full-length cDNA: (RESPONSIVE	1.51	1.92	3.48
247553_at	At5g60910	AGL8	MAD box containing protein NAP1-1 - like NAP1-1, Nicotiana tabacum,	1.73	0.62	0.23
247557_at	At5g61050	---	histone deacetylase-related / HD-related	0.57	0.21	0.35
247576_at	At5g61280	---	Remorin family protein;	0.07	0.52	0.80
247526_at	At5g61470	---	putative protein C2H2 zinc-finger protein, Petunia x hybrida, EMBL:ABC	1.45	0.23	0.67
247543_at	At5g61600	---	DNA binding protein - like DNA binding protein EREBP-4, encodes a me	1.88	2.08	2.51
247406_at	At5g62920	ARR6	response regulator 6 (ARR6) ; (RESPONSE REGULATOR 6); transcription	0.34	0.79	0.84
247326_at	At5g64110	---	peroxidase 70	0.71	0.36	1.21
247327_at	At5g64120	---	peroxidase 71	0.94	0.39	1.09
247240_at	At5g64660	U-box doma	putative protein strong similarity to unknown protein (emb|CAB89350.	1.44	1.35	2.17
247252_at	At5g64770	---	Encodes a root meristem growth factor (RGF).	0.57	1.99	0.27
247208_at	At5g64870	---	nodulin-like ;supported by full-length cDNA: Ceres:142026.	1.41	1.65	3.61
247125_at	At5g66070	---	RING/U-box superfamily protein; response to chitin	1.26	1.57	2.23
247128_at	At5g66110	---	atfp6-like protein; Heavy metal transport/detoxification superfamily pro	1.55	1.95	2.93
247047_at	At5g66650	---	putative protein	1.58	2.68	2.91
246984_at	At5g67310	CYP81G1	cytochrome P450	3.53	4.66	2.13
