# Supplementary material for: Organ-specific remodeling of the Arabidopsis transcriptome in response to spaceflight
Source: BMC Plant Biol. 2013 Aug 7;13:112. doi: 10.1186/1471-2229-13-112 (PMC3750915; doi:10.1186/1471-2229-13-112)
Supplement: Additional file 2 — Differential expression of 7-fold or greater in response to spaceflight among organs. Genes that show statistically significant (p < 0.01) differential expression of 7-fold or greater in response to spaceflight among organs are highlighted as bold text in the organ column in which that level of expression is displayed. [file 1471-2229-13-112-S2.pdf]

Probe ID	Atg number	Gene	Short Description	Fold	Fold	Fold
264564_at	At1g05290	---	CCT motif family protein	0.34	1.16	**12.04**
264643_at	At1g08990	PGSIP5	plant glycogenin-like starch initiation protein 5	**0.11**	1.55	0.97
264688_at	At1g09890	---	Rhamnogalacturonate lyase family protein	0.59	1.19	**9.99**
262810_at	At1g11710	---	putative salt-inducible protein	**0.13**	1.91	0.87
255937_at	At1g12610	DDF1	DWARF AND DELAYED FLOWERING 1, similar to CBF1	3.94	4.82	**24.25**
262857_at	At1g14930	---	major latex type2, Polyketide cyclase/dehydrase and lipid transport	**7.46**	0.95	1.28
256083_at	At1g20730	---	hypothetical protein	**0.12**	1.11	0.49
256084_at	At1g20750	---	RAD3-like DNA-binding helicase protein	**0.14**	0.81	0.27
264202_at	At1g22810	---	TINY-like transcription factor	4.53	0.36	**7.84**
245871_at	At1g26290	---	hypothetical protein	0.88	0.83	**0.11**
261984_at	At1g33760	---	TINY-like member of DREB subfamily of ERF/AP2 transcription factors	2.20	2.38	**10.27**
262724_s_at	At1g43330	---	PC-MYB2, putative similar to PC-MYB2	**0.12**	0.93	1.09
259453_at	At1g44090	GA20OX5	gibberelin 20-oxidase, putative	1.66	0.62	**0.12**
245749_at	At1g51090	---	proline-rich protein; Heavy metal transport/detoxification superfamily	0.99	2.93	**11.00**
259714_at	At1g60980	GA20OX5	putative gibberellin 20-oxidase	**0.10**	1.93	0.31
264733_at	At1g62170	---	phloem serpin-1, putative; Serine protease inhibitor (SERPIN) family	0.81	0.25	**7.06**
257453_at	At1g65130	---	Ubiquitin carboxyl-terminal hydrolase-related protein	5.24	0.86	**0.12**
261892_at	At1g80840	WRKY40	similar to WRKY pathogen-induced transcription factor	1.71	2.81	**7.46**
265748_at	At2g10620	---	putative Athila retroelement ORF1 protein	0.61	3.03	**11.08**
265397_at	At2g11090	---	transposable element gene	**0.10**	1.13	1.60
265889_at	At2g15130	---	Plant basic secretory protein (BSP) family protein; defense response	0.41	**8.82**	1.41
265589_at	At2g20170	---	hypothetical protein	0.59	1.36	**7.26**
245075_at	At2g23180	CYP96A1	putative cytochrome P450	1.37	**7.62**	0.56
265606_s_at	At2g25550	---	putative non-LTR retroelement reverse transcriptase	**0.11**	0.37	2.73
267644_s_at	At2g32880	---	TRAF-like family protein	0.84	1.51	**9.32**
265211_at	At2g36640	ATECP63	late embryogenesis abundant protein (AtECP63)	0.85	1.72	**12.73**
267361_at	At2g39920	---	HAD superfamily, subfamily IIIB acid phosphatase	0.75	0.89	**10.93**
260558_at	At2g43600	---	putative endochitinase	**0.12**	0.92	1.74
266753_at	At2g46990	IAA20	auxin-induced protein (IAA20) transcription factor	1.68	**0.14**	1.49
259044_at	At3g03430	---	pollen allergen Bra r II Calcium-binding EF-hand family protein	**7.84**	2.04	0.93
259331_at	At3g03840	---	putative auxin-induced protein similar to SAUR	0.76	1.40	**0.11**
259144_at	At3g10180	---	putative kinesin-like centromere protein	0.81	1.85	**0.12**
256289_s_at	At3g12230	scpl14	serine carboxypeptidase, (serine carboxypeptidase-like 14)	**0.10**	1.88	1.47
258438_at	At3g17230	---	invertase/pectin methylesterase inhibitor family protein	**9.19**	0.86	3.29
257950_at	At3g21780	UGT71B6	UDP-glucosyl transferase 71B6; abscisic acid glucosyltransferase	**7.78**	2.93	1.32
257842_at	At3g28390	PGP18	P-glycoprotein, putative; ATPase, transmembrane movement	**0.14**	0.45	3.94
256708_at	At3g30320	---	hypothetical protein	1.73	0.90	**0.11**
256571_at	At3g30730	---	hypothetical protein	**0.09**	1.13	0.97
252657_at	At3g44780	---	Cysteine proteinases superfamily protein	**0.13**	1.27	0.70
252460_at	At3g47230	---	transposable element gene	0.32	**7.11**	3.73
251824_at	At3g55090	---	ABC transporter - like; ATP-binding cassette-sub-family G-member 2	**0.14**	0.47	2.58
255141_at	At4g08420	---	transposable element gene; Mutator-like transposase family	0.54	0.72	**7.73**
255024_at	At4g09860	---	hypothetical protein	**8.17**	1.37	0.63
254733_at	At4g13760	---	putative polygalacturonase polygalacturonase, Pectin lyase-like	**0.12**	0.55	0.47
254735_at	At4g13810	AtRLP47	putative disease resistance protein Cf-4	1.00	0.55	**8.69**
253910_at	At4g27290	---	putative receptor-like kinase SFR2	**7.01**	1.27	0.57
250561_at	At5g08030	---	glycerophosphodiester phosphodiesterase - like protein	**9.65**	0.67	1.00
250071_at	At5g18000	VDD	VERDANDI, putative transcription factor, reproductive meristem	0.78	1.53	**8.69**
246669_at	At5g29958	---	galactinol synthase, putative	1.05	1.59	**0.13**
246656_at	At5g35240	---	transposable element gene	0.47	2.73	**10.93**
249673_at	At5g35920	CYP79A4P	cytochrome P450-like	0.52	1.88	**0.11**
249664_at	At5g36810	---	putative protein histone stem-loop binding protein	3.18	1.16	**10.56**
249607_at	At5g37280	---	putative protein RING-H2 finger protein RHA1b	**9.92**	0.90	2.17
249141_at	At5g43200	---	Zinc finger, C3HC4 type (RING finger) family protein	0.81	**0.12**	1.15
248645_at	At5g49150	GEX2	(GAMETE EXPRESSED 2)	1.09	0.51	**7.52**
248514_s_at	At5g50510	---	unknown protein	0.80	0.47	**0.13**
247789_at	At5g58680	---	ARM repeat superfamily protein	1.83	**8.57**	**15.56**
247576_at	At5g61280	---	Remorin family protein;	**0.07**	0.52	0.80
