# Supplementary material for: Organ-specific remodeling of the Arabidopsis transcriptome in response to spaceflight
Source: BMC Plant Biol. 2013 Aug 7;13:112. doi: 10.1186/1471-2229-13-112 (PMC3750915; doi:10.1186/1471-2229-13-112)
Supplement: Additional file 3 — Cellular remodelling and hormone signaling associated genes differentially expressed genes in response to spaceflight. The hierarchical clustering of 158 genes with statistically significant (p < 0.01) differential expression in the spaceflight environment by at least 1.9-fold in at least one of the three organs, and which have an association with cell wall remodeling and cell expansion, pathogen or wounding responses, and growth hormone signal transduction. The graphic representation of gene expression patterns is annotated with the corresponding AtG number, gene name, and notes associated with that gene’s functional association. [file 1471-2229-13-112-S3.pdf]

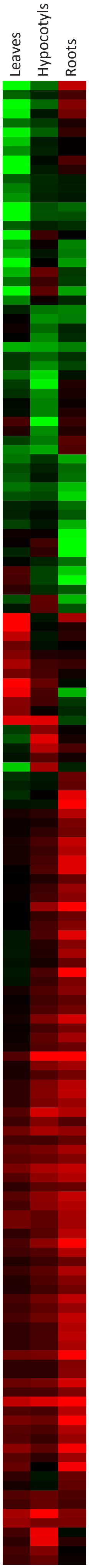

At3g28390 | PGP18 | P-glycoprotein, putative P-GLYCOPROTEIN 18; ATPase, coupled to transmembrane movement of substances
At3g29030 | EXPA5 | expansin At-EXP5 identical to expansin At-EXP5 EXPANSIN A5
At3g55090 | --- | ABC transporter - like protein ATP-binding cassette-sub-family G-member 2, Mus musculus, EMBL:AF140218
At1g34170 | ARF13 | auxin response factor 13
At2g43860 | --- | putative polygalacturonase; Pectin lyase-like superfamily protein
At1g20480 |4CL-like4 | AMP-dependent synthetase and ligase family protein; phenylpropanoid metabolic process
At3g62710 | --- | beta-D-glucan exohydrolase-like protein exhydrolase II - Zea mays, EMBL:AF064707
At1g16530 | ASL9 | ASYMMETRIC LEAVES 2 LIKE 9
At2g43600 | --- | putative endochitinase
At2g21490 | LEA | DEHYDRIN LEA putative dehydri n
At4g28250 | ATEXPB3 EXPB3 ARABIDOPSIS THALIANA EXPANSIN B3 | putative beta-expansin; plant-type cell wall loosening
At1g44800 | --- | nodulin MtN21 /EamA-like transporter family protein; membrane protein binidng
At5g62920 | ARR6 | response regulator 6 ARR6 ; RESPONSE REGULATOR 6; transcription regulator/ two-component response regulato r
At4g13760 | --- | putative polygalacturonase polygalacturonase, Pectin lyase-like superfamily protei n
At1g78440 | ATGA2OX1 | gibberellin 2- oxidase gibberellin 2-oxidase 1; gibberellin 2-beta-dioxygenase
At3g51660 | --- | LS1-like protein AT-LS1 product - Tautomerase/MIF superfamily protein; INVOLVED IN: inflammatory response
At1g08990 | PGSIP5 | plant glycogenin-like starch initiation protein 5
At1g34510 | --- | peroxidase ATP13a, putative; assoc with Pi diffeciency and cell wall remodeling
At3g54580 | --- | extensin precursor -like protein extensin precursor,EXT10, root specific, Proline-rich extensin-like famil y
At3g58300 | --- | Arabidopsis phospholipase-like protein PEARLI 4 famil y
At2g24450 | FLA3 | FASCICLIN-LIKE ARABINOGALACTAN PROTEIN 3 PRECURSOR
At2g38540 | LP1 | calmodulin binding; putative nonspecific lipid-transfer protei n
At1g60980 | ATGA20OX4 | putative gibberellin 20-oxidase
At1g64380 | --- | member of the DREB subfamily A-6 of ERF/AP2 transcription factor
At1g56650 | PAP1 | PRODUCTION OF ANTHOCYANIN PIGMENT 1 similar to anthocyanin2 An2; MYB75
At4g12550 | AIR1 | putative cell wall-plasma membrane disconnecting CLCT protein AIR1A ;; lipid binding
At2g37130 | --- |PER21 putative peroxidase ATP2a
At1g02950 | ATGSTF4 | glutathione-S-transferase
At5g22430 | --- | Pollen Ole e 1 allergen and extensin family protei n
At1g48100 | --- | polygalacturonase PG1, putative; Pectin lyase-like superfamily protein; FUNCTIONS IN: polygalacturonase acti vity
At3g24650 | ABI3 | ABA INSENSITIVE 3; DNA binding / basal transcription repressor/ transcription activator/ transcription fact or1992
At2g42430 | LBD16 | LATERAL ORGAN BOUNDARIES-DOMAIN 16 LOB-domain contains one auxin-responsive element AuxRE.
At3g52640 | --- | Zn-dependent exopeptidases superfamily protei n
At5g64110 | --- | peroxidase 70
At5g64120 | --- | peroxidase 71
At2g43620 | --- | putative endochitinase
At2g46990 | IAA20 | auxin-induced protein IAA20 INDOLE-3-ACETIC ACID INDUCIBLE 20; transcription factor
At2g35700 | ERF38 | ethylene-responsive transcription factor ; putative AP2 domain transcription factor; ERF FAMILY PROTEIN 38;
At3g51560 | --- | disease resistance-like protein TMV resistance protein N, Nicotiana glutinosa, PIR:A54810
At5g35940 | --- | putative protein myrosinase-binding protein-like; also similar to jasmonate inducible protein-like
At2g43700 | --- | Concanavalin A-like lectin protein kinase famil y
At5g52860 | --- | ABC transporter-like protein
At4g08780 | --- | peroxidase C2 precursor like protein peroxidase EC 1.11.1.7 C2 precursor - Armoracia rusticana,PID:d1014866
At2g44810 | DAD1 | DEFECTIVE ANTHER DEHISCENCE 1; phospholipase A1/ triacylglycerol lipase putative triacylglycerol lipase
At5g20340 | BG5 | beta-1,3-glucanase bg5
At4g27890 | --- | HSP20-like chaperones
At4g23496 | SP1L5 | SPIRAL1-LIKE5 Regulates cortical microtubule organization. anisotropic cell expansion
At1g05650 | --- | Pectin lyase-like superfamily protein; FUNCTIONS IN: polygalacturonase activity
At5g26070 | --- | extensin - like protein pAP8 protein, Nicotiana tabacum, PIR:S33159
At5g29958 | --- | galactinol synthase, putative
At3g03840 | --- | putative auxin-induced protein similar to SAUR
At4g34580 | COW1 | putative protein SEC14 protein, CAN OF WORMS1; root epidermal cell differentiation, root hair cell tip gro wth, transport
At5g60910 | AGL8 | MAD box containing protein NAP1-1 - like NAP1-1, Nicotiana tabacum, agamous-like 8; transcription factor
At1g44090 | ATGA20OX5 | gibberelin 20-oxidase, putative
At4g16350 | CBL6 | CALCINEURIN B-LIKE PROTEIN 6; calcium ion binding
At5g64770 | --- | Encodes a root meristem growth factor RGF.
At2g34830 | WRKY35 WRKY DNA-binding protein 35; transcription factor | embryo development ending in seed dormancy
At3g17230 | --- | invertase/pectin methylesterase inhibitor family protein;
At1g14930 | --- | major latex homologue type2, Polyketide cyclase/dehydrase and lipid transport superfamily protein
At1g10300 | --- | putative GTP-binding protein similar to S. cerevisiae Lpg15p
At2g47000 | ABCB4 | ATP BINDING CASSETTE SUBFAMILY B4 ATP catabolic process, ATPase activity, ATPase activity,
At5g20150 | SPX1 | ids4-like protein, SPX DOMAIN GENE 1 positive regulation of cellular response to phosphate starvation in roots
At4g37990 | ELI3-2 | cinnamyl-alcohol dehydrogenase ELI3-2 ; ELICITOR-ACTIVATED GENE 3-2; aryl-alcohol dehydrogenase
At1g72890 | --- | Disease resistance protein TIR-NBS class
At5g03430 | --- | pollen allergen Bra r II Calcium-binding EF-hand family protei n
At5g26080 | --- | extensin - like protein hydroxyproline-rich glycoprotein precursor, Nicotiana tabacum, PIR:S06733
At3g15500 | ANAC055 | putative jasmonic acid regulatory protein; Encodes an ATAF-like NAC-domain transcription factor
At2g29490 | ATGSTU1 GLUTATHIONE S-TRANSFERASE TAU 1; glutathione transferase | putative glutathione S-transferase
At4g00680 | ADF8 | putative actin-depolymerizing factor ; ACTIN DEPOLYMERIZING FACTOR 8; actin binding
At1g61490 | --- | S-locus lectin protein kinase family protein; receptor kinase
At3g04100 | AGL57 | AGAMOUS-like 57 ; putative SRF-type transcription factor
At1g43040 | --- | auxin-induced protein, putative
At3g47540 | --- | endochitinase-like protein BASIC ENDOCHITINASE CHB4 PRECURSOR - cell wall macromolecule catabolic process
At5g58830 | --- | subtilisin-like serine protease contains similarity to prepro-cucumisin GI:807698 from [Cucumis melo]
At1g69730 | --- | Wall-associated kinase family protein;
At1g76470 | --- | putative cinnamoyl-CoA reductase; lignin biosynthes
At2g37030 | --- | SAUR-like auxin-responsive protein family
At2g40740 | WRKY55; transcription factor | putative WRKY-type III DNA binding protein
At1g35140 | PHI-1 | EXL1, EXORDIUM LIKE 1, PHI-1, PHOSPHATE-INDUCED 1, located plant cell wall
At5g24590 | TIP | NAC2-like protein TCV-INTERACTING PROTEIN; transcription coactivator/ transcription factor
At1g53840 | ATPME1 | encodes a pectin methylesterase; cell wall modification
At3g28340 | GATL10 | Galacturonosyltransferase-like 10; polygalacturonate 4-alpha-galacturonosyltransferase
At3g50060 | MYB77 | R2R3-MYB transcription factor ; assoc w lateral root development
At3g44260 | CAF1 | CAF1; CCR4-associated factor 1-like protein; wounding, induced 3x in treatment that induces cell wall appos ition Guan 2004
At1g73540 | atnudt21 Arabidopsis thaliana Nudix hydrolase homolog 21; hydrolase | nudix hydrolase 21-like
At5g44350 | --- | ethylene-regulated nuclear protein ERT2-like protein
At2g27080 | --- | a LEA
At3g62720 | XT1 | alpha galactosyltransferase-like protein alpha galactosyltransferase - XYLOSYLTRANSFERASE 1; root hair elon gation
At1g51090 | --- | proline-rich protein, putative ; Heavy metal transport/detoxification superfamily protei n
At2g40750 | WRKY54; transcription factor | member of WRKY Transcription Factor; Group III
At2g31880 | --- | putative receptor-like protein kinase ; Encodes a putative leucine rich repeat transmembrane protein
At5g57560 | TCH4 | XTH22 TCH4 protein gb|AAA92363.1 ; supported by cDNA: gi_14194112_gb_AF367262.1_AF367262
At4g08950 | EXO | EXORDIUM putative phi-1-like phosphate-induced protein ;exordium; plant-type cell wall, response to brassi nosteroid stimulus
At2g47190 | MYB2 | MYB transcription factor Atmyb2MYB DOMAIN PROTEIN 2; DNA binding / calmodulin binding /
At5g37770 | TCH2 | CALMODULIN-RELATED PROTEIN 2, TOUCH-INDUCED TCH2 ;TOUCH 2; calcium ion binding
At2g36640 | ATECP63 | late embryogenesis abundant protein AtECP63 ; EMBRYONIC CELL PROTEIN 63
At4g34150 | --- | Calcium-dependent lipid-binding CaLB domain family protei n
At3g49530 | anac062 | NAC2-like protein NAC2 - Arabidopsis NAC domain containing protein 62; transcription factor
At3g28180 | ATCSLC04 CELLULOSE-SYNTHASE LIKE C4; cellulose synthase/ transferase, transferring glycosyl groups | glycosyl group s
At3g56400 | WRKY70; transcription factor/ transcription repressor |
At3g09530 | ATEXO70H3 | exocyst subunit EXO70 family protein H3; protein binding
At5g58430 | ATEXO70B1 exocyst subunit EXO70 family protein B1; protein binding | leucine zipper-containing protei n
At5g35735 | --- | Auxin-responsive family protein
At3g45970 | ATEXLA1 | EXPL1 ARABIDOPSIS THALIANA EXPANSIN-LIKE A1 putative protein cim1 induced allergen
At5g58680 | --- | ARM repeat superfamily protein
At5g17850 | --- | potassium-dependent sodium-calcium exchanger - like protein cone sodium-calcium potassium exchanger NCKX,
At1g19180 | JAZ1 | JAZ1 is a nuclear-localized protein involved in jasmonate signaling
At2g40000 | HSPRO2 | HS1 PRO-1 2-like protein defense response to bacterium
At5g45340 | CYP707A3; +-abscisic acid 8'-hydroxylase/ oxygen binding | cytochrome P450
At4g31800 | WRKY18; transcription factor | Pathogen-induced transcription factor
At5g05410 | DREB2A; DNA binding / transcription activator/ transcription factor |
At5g40170 | AtRLP54 | disease resistance - like protein resistance gene Cf-4, Lycopersicon hirsutum, EMBL:LHJ002235
At5g04340 | ZAT6 ZINC FINGER OF ARABIDOPSIS THALIANA 6; root development
At2g27500 | --- | putative beta-1,3-glucanase ;Glycosyl hydrolase superfamily protein; cation binding, hydrolase activity
At1g60190 | PUB19 | Encodes PUB19, a plant U-box armadillo repeat protein. Involved in salt inhibition of germination
At5g61600 | --- | DNA binding protein - like DNA binding protein EREBP-4, encodes a member of the ERF ethylene response factor
At1g15010 | --- | unknown, seems involved in defense response to fungus
At1g28480 | GRX480 | member of the glutaredoxin family, some assoc w cell expansion
At1g21910 | --- | TINY-like protein; encodes a member of the DREB subfamily A-5 of ERF/AP2 transcription factor famil y
At3g61190 | BAP1 | BON ASSOCIATION PROTEIN 1; phospholipid binding / protein binding down regulation of defense response
At5g54490 | PBP1 | PINOID-BINDING PROTEIN 1; calcium ion binding / protein binding
At2g15880 | --- | Leucine-rich repeat LRR family protein; structural constituent of cell wall
At2g35930 | PUB23 | PLANT U-BOX 23; U-box domain containing E3 ubiquitin ligase
At2g33580 | --- | Protein kinase superfamily protein; kinase activity; cell wall macromolecule cataboli c
At1g80840 | WRKY40; transcription factor | similar to WRKY transcription factor; Pathogen-induced transcription factor
At5g22250 | --- | CAF1B CCR4-associated factor-like protein
At4g18880 | AT-HSFA4A | heat shock transcription factor - like protein heat shock transcription factor, Zea mays, PIR2:S61448
At5g59820 | RHL41 | zinc finger protein Zat12 ;supported by full-length cDNA: RESPONSIVE TO HIGH LIGHT 41;
At1g32640 | MYC2 | Encodes a MYC-related transcriptional activator; jasmonate insensitive; ZBF1
At5g66070 | --- | RING/U-box superfamily protein; response to chitin
At4g25810 | XTR6 | XTH23 xyloglucan endo-1,4-beta-D-glucanase XTR-6 XYLOGLUCAN ENDOTRANSGLYCOSYLASE 6;
At5g66110 | --- | atfp6-like protein; Heavy metal transport/detoxification superfamily protei n
At2g30020 | --- | putative protein phosphatase 2C; Encodes AP2C1. Belongs to the clade B of the PP2C-superfamily.
At5g64870 | --- | nodulin-like ;supported by full-length cDNA: Ceres:142026.
At2g05940 | --- | RIPK - Encodes a receptor-like cytoplasmic kinase that phosphorylates the host target RIN4
At1g17380 | JAZ5 | jasmonate-zim-domain protein 5
At1g33760 | --- | TINY-like protein; encodes a member of the DREB subfamily A-4 of ERF/AP2 transcription factor famil y
At1g66400 | --- | Encodes a calmodulin-like protein. Regulates nitric oxide levels and transition to flowering
At5g49520 | WRKY48; transcription factor | WRKY48 is a stress- and pathogen-induced transcriptional activator
At2g41640 | --- | Glycosyltransferase family 61, cell wall - pectins
At1g13260 | RAV1 | transcription factor DNA-binding protein RAV1, Encodes an AP2/B3 domain transcription factor
At1g12610 | DDF1 DWARF AND DELAYED FLOWERING 1 | transcriptional activator CBF1, putative similar to transcriptional activator CBF1
At5g47220 | ERF2 | ethylene responsive element binding factor 2 ATERF2 ETHYLENE RESPONSIVE ELEMENT BINDING FACTOR 2
At5g42380 | CML37 | CALMODULIN LIKE 37; calcium ion binding
At1g77640 | --- | encodes a member of the DREB subfamily A-5 of ERF/AP2 transcription factor famil y
At1g72920 | --- | virus resistance protein, Toll-Interleukin-Resistance TIR domain family protei n
At3g23250 | MYB15 | myb-related transcription factor, auxin, wounding
At1g44830 | --- | encodes a member of the DREB subfamily A-5 of ERF/AP2 transcription factor famil y
At3g09040 | --- | unknown pentatricopeptide repeat-containing protein, but similar motif found in PHIP, which is assoc w cell plate
At5g26920 | CBP60G | calmodulin-binding - like protein calmodulin-binding protein TCB60,
At4g32800 | --- | transcription factor TINY homolog encodes a member of the DREB subfamily A-4 of ERF/AP2 transcription factor family
At2g44080 | ARL ARGOS-LIKE | Encodes ARL, a gene similar to ARGOS involved in cell expansion-dependent organ growth
At1g17870 | EGY3 | ETHYLENE-DEPENDENT GRAVITROPISM-DEFICIENT AND YELLOW-GREEN-LIKE 3; S2P-like putative metalloprotease
At2g34600 | JAZ7 | JASMONATE-ZIM-DOMAIN PROTEIN 7; protein TIFY 5B responds to chitin and jasmonate
At2g16720 | MYB7 | putative MYB family transcription factor response to salt and salicylic acid
At1g54470 | RPP27 | Encodes a Cf-like gene in Arabidopsis that confers downy mildew resistance
At1g59725 | --- | DNAJ heat shock family protein; chaperone, putative similar to chaperone GI:3452219 from [Trypanosoma cruzi]
At1g06160 | ORA59 | OCTADECANOID-RESPONSIVE ARABIDOPSIS AP2/ERF 59; ethylene response factor,
At4g09460 |ATMYB6 |myb6 DNA-bindi ng protein response to gibberellin stimulu s, response to jasmonic acid stimulu s,
