# Supplementary material for: Organ-specific remodeling of the Arabidopsis transcriptome in response to spaceflight
Source: BMC Plant Biol. 2013 Aug 7;13:112. doi: 10.1186/1471-2229-13-112 (PMC3750915; doi:10.1186/1471-2229-13-112)
Supplement: Additional file 4 — RT-qPCR primers and probes. The forward and reverse primers used for RT-qPCR anaylse of DDF1, DREB2A, TCH4, JAZ7, ELIP1, and the UBQ11 control. Primers and probes were designed with Primer Express software and supplied by Applied Biosystems. [file 1471-2229-13-112-S4.pdf]

gene	AtG	Forward (5'→3')	Reverse (5'→3')	Probe (dye 5'→3'quencher)
DDF1	At1g12610	CAGAGAACCGACGCACCAA	GCACGCGCTGCCATATCT	6FAM-<br>CCGCATTTGGCTCGGGACTTATCC<br>-TAMRA
DREB2A	AT5G05410	ACAGTGTTGCCAACGGTTCA	TGAGGCTTTGTAGCGGATCA	6FAM-<br>ACAGGCCCGAGAGTCAACAAAGTGGTT<br>-TAMRA
TCH4	At5g57560	TGGAACCCACAAAGAATCATTTT	GTGCCTAGAGACTCCATGTTCTTG	6FAM-<br>ACCGTCGATGGAACTCCGATCAGAGA<br>-TAMRA
JAZ7	At2g34600	CTGCGACAAGCCTTTACTCAATT	AGAAGTAAGAAGGCGAAGTTCCAA	6FAM-<br>CAAAGAGATGGAGATGCAAACAAAATGCG<br>-TAMRA
ELIP1	At3g22840	TCGGTACAACAGCGATCTTGAC	ACTCAACGCTTATGCCCTTGA	6FAM-<br>CTTGCGTCGCTTGTGCCGCTT<br>-TAMRA
UBQ11	At4g05050	AACTTGAGGACGGCAGAACTTT	GTGATGGTCTTTCCGGTCAAA	VIC-<br>CAGAAGGAGTCTACGCTTCATTTGGTCTTGC<br>-TAMRA
